# What Impact Does Net Zero Action on Road Transport and Building Heating Have on Exposure to UK Air Pollution?

**DOI:** 10.1021/acs.est.4c05601

**Published:** 2025-01-10

**Authors:** Nosha Assareh, Andrew Beddows, Gregor Stewart, Mike Holland, Daniela Fecht, Heather Walton, Dimitris Evangelopoulos, Dylan Wood, Tuan Vu, David Dajnak, Christian Brand, Sean David Beevers

**Affiliations:** † Environmental Research Group, School of Public Health, Imperial College London, Sir Michael Uren Biomedical Engineering Hub, White City Campus, 80 Wood Lane, London W12 0BZ, United Kingdom; ‡ MRC Centre for Environment and Health, School of Public Health, Imperial College London, Sir Michael Uren Biomedical Engineering Hub, White City Campus, 80 Wood Lane, London W12 0BZ, United Kingdom; § Ecometrics Research and Consulting, Reading RG8 7PW, United Kingdom; ∥ School of Public Health, Faculty of Medicine, Imperial College London, White City Campus, 80 Wood Lane, London W12 0BZ, United Kingdom; ⊥ NIHR HPRU in Environmental Exposures and Health, School of Public Health, Imperial College London, Sir Michael Uren Biomedical Engineering Hub, White City Campus, 80 Wood Lane, London W12 0BZ, United Kingdom; # Transport Studies Unit, University of Oxford, South Parks Road, Oxford OX1 3QY, United Kingdom

**Keywords:** net zero, air pollution modeling, exposure, electric vehicles, building heating

## Abstract

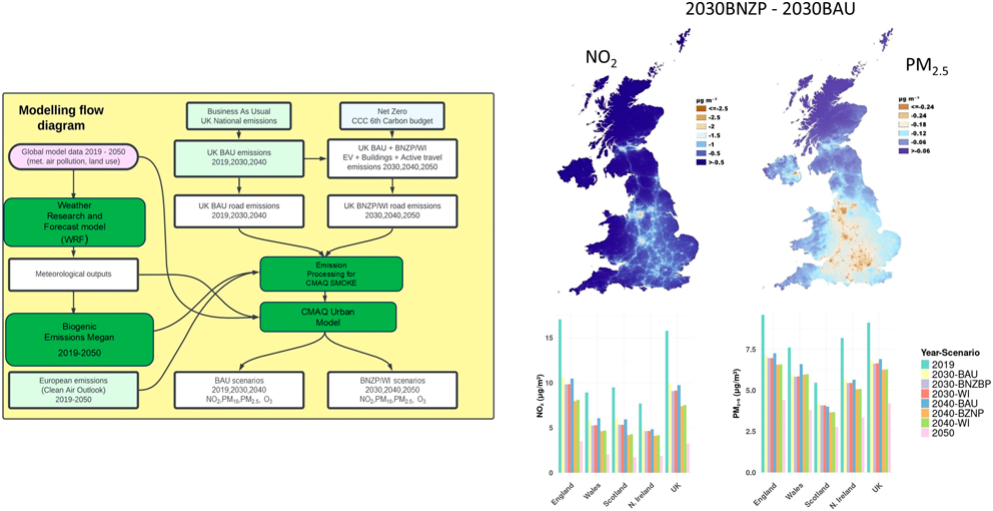

This study explores the cobenefits of reduced nitrogen dioxide (NO_2_), ozone (O_3_), and particulate matter (PM), through net zero (NZ) climate policy in the UK. Two alternative NZ scenarios, the balanced net zero (BNZP) and widespread innovation (WI) pathways, from the UK Climate Change Committee’s Sixth Carbon Budget, were examined using a chemical transport model (CTM). Under the UK existing policy, Business as Usual (BAU), reductions in NO_2_ and PM were predicted by 2030 due to new vehicle technologies but plateau by 2040. The BNZP and WI scenarios show further reductions particularly by 2040, driven by accelerated electric vehicle (EV) uptake and low-carbon heating in buildings, with the building contribution to PM reduction being 2–3 times greater than road transport. The results demonstrate that the NZ transition to EVs (cars and vans) reduces both exhaust and nonexhaust emissions, as well as reducing traffic volumes. O_3_ trends are complex with a small overall increase by 2030 and a decrease by 2040. Although uncertain, 2050 predictions of BNZP showed important additional air pollution benefits. Our findings highlight the efficacy of NZ strategies, providing insights for UK and international policymakers interested in the air pollution cobenefits of climate policy.

## Abstract 2

We predicted air quality benefits of NZ policy for buildings, transport, and active travel in 2030 and 2040 compared with the existing UK policy, predicting further NZ benefits in 2050.

## Introduction

The impact of climate change on various aspects of the environment and public health is a topic of growing concern, highlighted by increasing evidence of its effects on air pollution, indoor environments, ecosystems, and social dynamics.^1^ Anthropogenic greenhouse gas (GHG) emissions are the primary drivers of these changes, also contributing to the release of harmful air pollutants. Climate change exacerbates air pollution through elevated temperatures and shifting weather patterns, affecting the dispersion of particulate matter and intensifying the formation of temperature-dependent secondary pollutants such as ozone.^2^ Moreover, indirect effects such as increased wildfire frequency further deteriorate air quality and amplify related health risks, including respiratory and cardiovascular diseases.^3−7^

The Intergovernmental Panel on Climate Change (IPCC) emphasized the need to significantly reduce GHG emissions and achieve net zero (NZ) emissions by the early 2050s to prevent the most severe consequences of climate change.^8^ While many policies aimed at reducing GHGs target common emission sources like fossil fuel combustion, these measures can result in both cobenefits and trade-offs affecting air quality.^9^ In response, countries worldwide have adopted a range of policies aimed at combating climate change by 2050, based on commitments under international accords such as the 1992 United Nations Framework Convention on Climate Change, the 1997 Kyoto Protocol, and the 2015 Paris Agreement.^10−13^

In 2019, the UK became the first major economy to legislate a commitment to achieving NZ emissions by 2050.^14^ This NZ commitment is a comprehensive strategy to either completely eliminate GHG emissions or balance these emissions through equivalent removals from the atmosphere, encompassing direct emission reductions across all sectors and the use of carbon offsets such as afforestation and carbon capture and storage (CCS).^15^ The UK’s approach updates and intensifies previous climate commitments under the Climate Change Act, which aimed for an 80% reduction in GHG emissions from 1990 levels by 2050.

Several studies have investigated UK’s different emission reduction policies, both within and beyond the scope of NZ strategies in the UK, to assess their impact on air quality as well as the associated health cobenefits.^16−20^ While these studies have presented valuable insights, our study aims to expand upon these efforts by examining the implications of a set of future policies. Specifically, we focus on those currently agreed upon in the Business as Usual (BAU) scenario and those aligned with the Climate Change Committee’s (CCC) NZ targets, emphasizing their potential to enhance air quality and public health. Unlike most of the previous studies, which either rely on base year emissions or concentrations remaining the same into the future,^20,21^ or extrapolated using historical trends for future projections,^22^ our approach introduces a more realistic BAU scenario where future emissions were based on existing measures and policies, developed through discussions with the UK Government and CCC. By comparing air quality outcomes, under these BAU scenarios, with improvements anticipated from the adoption of NZ policies, our research underscores more realistic benefits of pursuing NZ strategies. This in turn links the results directly to UK air quality and legally binding NZ policy and hopefully to more impactful research, although we accept that using current policies for BAU future forecasts comes with its own uncertainties. This comparative analysis extends to include interim years 2030 and 2040, to underscore the progressive impact of these policies over time. Prompt assessment of the impacts of these policies on air quality and health is crucial for maximizing short- and long-term benefits. We have used high-resolution numerical simulations with a coupled chemical transport model (CTM) and a local scale dispersion road model to predict UK-wide air pollutant concentrations. It is also important to note that while this study primarily focuses on air quality impacts, it is part of a broader research project, with other publications forthcoming that will cover health impact analyses, inequalities, cost-benefit assessments, and indoor air quality, contributing to a more comprehensive understanding of the multifaceted effects of NZ policies.

## Methods

### Overview

**Figure 1:**
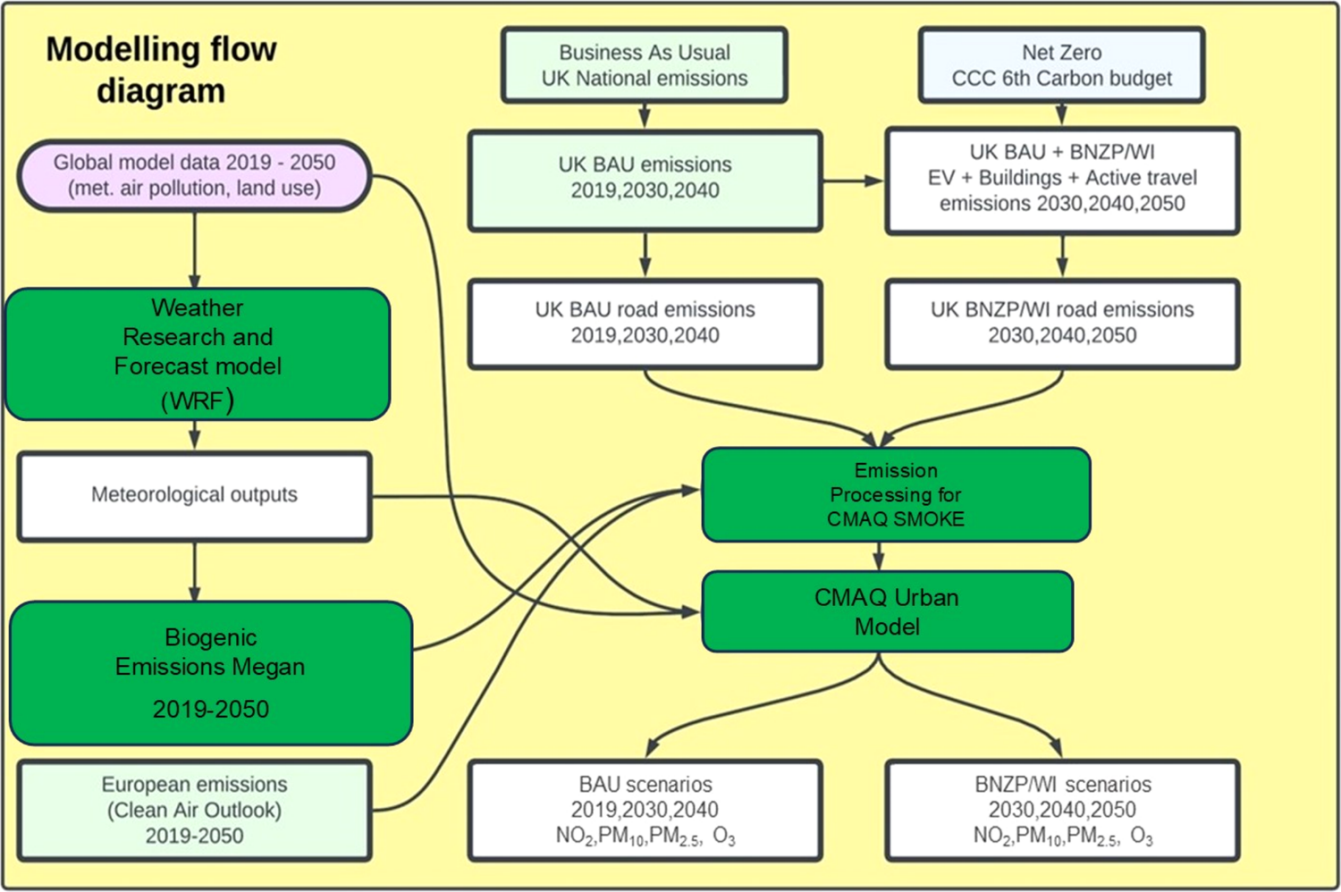
Modeling Flow diagram.

The modeling framework is shown in Figure 1, which illustrates the inputs, models, scenarios, and outputs. It covers regional and UK emissions, meteorological data, and biogenic sources, along with projected emission scenarios, either BAU or NZ alternatives, all feeding into the air quality model and resulting in predicted air quality concentrations. The BAU and NZ scenarios can then be compared to estimate cobenefits. Each component is explained in detail in the following sections.

### Scenarios

The BAU scenario, which projects for the years 2019, 2030, and 2040, reflects existing UK environmental policies and commitments prior to the NZ 2050 legislation. These include measures on Industrial Emissions, Euro standards for vehicles, and energy projections by the Department for Business, Energy and Industrial Strategy,^23^ but exclude the effects of UK’s Clean Air Strategy,^24^ which typically address small and localized air quality problems in cities. Given that the Clean Air Strategy initiatives primarily target vehicle emissions and that the impact of low-emission zones diminishes over time as older vehicles are replaced by newer, compliant models, we have concentrated on the broader shifts in the BAU scenario. This approach provides a cautious yet realistic projection of future emissions, serving as a realistic benchmark with which to compare the NZ-aligned strategies. Subsequently, we considered BAU plus alternative NZ scenarios for buildings and road transport, including active travel, targeting the years 2030 and 2040. For residential, commercial, and public buildings we have drawn upon the CCC’s Sixth Carbon Budget-balanced NZ pathway (BNZP),^25^ including energy efficiency measures, behavior change, and a wide mix of technologies such as low-carbon district heat networks, air and ground source heat pumps, resistive and storage heating, solar thermal, hydrogen boilers, and hydrogen hybrid heat pumps. BNZP also requires the removal of biomass burning as well as the benefits of indoor air quality exposure from switching from gas to electric cooking. The BNZP’s four priorities are to deliver on the Government’s energy efficiency plans, to scale up the market for heat pumps, to expand the rollout of low-carbon district heat networks, and to prepare for a potential role for hydrogen in heat energy production, exemplified by the UK Governments target of 600k heat pump installations per annum.^26^ For road transport, we included both BNZP and widespread innovation (WI) scenarios for 2030 and 2040. The BNZP represents a swifter transition to electric vehicles (EV) and reducing vehicle kilometers (VKM) compared to the BAU scenario. The more ambitious WI scenario assumes accelerated advancements in battery technology, leading to more affordable electric vehicles and a faster adoption rate than that in the BNZP scenario. The comprehensive assumptions and methodologies underpinning the emission calculations for each scenario are detailed in Section S1. Note that for the year 2050, the target year for achieving net zero emissions, we combined BNZP assumptions for road transport, residential, and nonresidential buildings which are outlined in detail in Section S1, and for all the other sources emissions were based on the European Commission’s Second Clean Air Outlook v2021^27^ projections.

### Emissions

European emissions of NO_*x*_, CO, PM_10_ (PM with an aerodynamic diameter <10 μm), PM_2.5_ (PM < 2.5 μm), SO_2_, HCl, VOCs, and NH_3_ were acquired from the European Monitoring and Evaluation Programme (EMEP) Centre on Emission Inventories and Projections^28^ for 2019 and summarized as a set of 50 km grids. The EMEP emissions were classified into 11 Selected Nomenclature for Air Pollution (SNAP) source types (Table S1), and the anthropogenic emissions were further processed into hourly gridded chemical species using methods developed in the US-EU “Air Quality Modeling Evaluation International Initiative” (AQMEII) project.^29,30^ Future European emissions for each nation-state (see % change between 2019 and 2030 to 2050 in Table S1) were taken from the European Commission’s Second Clean Air Outlook v2021,^27^ providing total emissions for all pollutants, by snap sector, for each nation from now until 2050. The EU’s Second Clean Air Outlook Report outlines a comprehensive strategy aimed at reducing air pollutants across the EU, leveraging existing legislation and policies, including the National Emission Reduction Commitments Directive (NEC Directive) and sector-specific measures that target major sources of air pollution. While we have developed detailed future emissions scenarios for the UK based on the BAU, BNZP, and WI pathways, applying a single emission scenario for the rest of Europe is a limitation of our study and is primarily driven by the availability of consistent, comprehensive future emissions data for European countries and the need to maintain a manageable scope for our modeling efforts.

UK 2019 anthropogenic emissions of NO_*x*_, CO, PM_10_, PM_2.5_, SO_2_, HCl, VOCs, and NH_3_, excluding roads and buildings, were taken from the National Atmospheric Emissions Inventory (NAEI).^31^ These emissions were also categorized into SNAP sectors and subsequently processed into hourly 2 km gridded species for detailed spatial analysis. The UK BAU emission forecasts between 2019 and 2030/2040 were taken from DEFRA’s projections.^32^ For London, 2019 emissions were taken from the London Atmospheric Emissions Inventory (LAEI)^33^ and for 2030BAU projections were based on commitments made in the London Environment Strategy,^34^ and in 2040BAU, followed the UK’s projections between 2030 and 2040. The residential, commercial, and public building emissions forecasts for 2030, 2040, and 2050BNZP scenarios were based upon behavioral changes, energy efficiency measures, and low-carbon sources described in the Overview section above and were the same as in the WI scenario.

UK vehicle emissions were calculated using Imperial College London’s established road emissions model^20,35^ – under the BAU scenarios, incorporating vehicle fleet composition and traffic projections from the UK NAEI for 2020.^31^ In London, the BAU scenarios used data from the Greater London Authority’s (GLA) LAEI 2019 projections to 2030.^33^ In addition, for the BNZP and WI scenarios, vehicle emissions were estimated using vehicle kilometers and fleet forecasts from the CCC’s BNZP scenario.^25^ Total UK emissions for each scenario and SNAP sector, along with specific details of the UK emissions estimation methodology, are summarized in Table S3 and Section S1, respectively.

### Meteorology and Air Pollution Modeling Methods

For our assessment, we used the CMAQ-urban model,^20^ a novel approach and relevant to UK air quality policy and target setting. CMAQ-urban is a combination of the Weather Researching and Forecasting (WRF V4.2) meteorological model,^36^ the USEPA CMAQ model (V5.4),^37^ and the ADMS-Roads model.^38^ This hybrid modeling approach enables us to produce air pollution forecasts at scales from a broad 50 km grid covering Europe, to a more detailed 10 and 2 km grid across the UK, and down to highly localized predictions every 20 m near roads. The modeling domains (Figure S2), along with detailed model configurations, are provided in Section S2. The CMAQ model was operated over the same domain as the WRF model, which output concentrations of key air pollutants.

The lateral boundary conditions for the 2019 WRF simulation were taken from the National Centres for Environmental Prediction (NCEP) Final Operational Model Global Tropospheric Analyses (FNL) with a 6-h interval and 0.25° × 0.25° grid resolution.^39^ Meteorological fields for future years (2030, 2040, and 2050) were driven by boundary conditions from a bias-corrected global data set based on ensemble outputs from the Coupled Model Intercomparison Project Phase 6 (CMIP6) and the European Centre for Medium-Range Weather Forecasts Reanalysis 5 (ERA5), under the SSP2-4.5 socioeconomic pathway.^40^ To further validate the use of bias-corrected CMIP6 data for simulations of future years, we conducted a sensitivity test by initializing the WRF model with these data for the base year of 2019 and comparing the results with those derived from FNL data. Since 2019 is a future projection within the CMIP6 framework, its performance is understandably lower compared to FNL data in terms of interannual variability and correlation coefficients.^41^ However, in terms of overall mean and bias, the performance was acceptable, revealing relatively small differences in the annual means of key parameters across the UK, such as temperature (0.3 °C) and wind speed (0.5 m s^–1^), confirming the efficacy of using CMIP6 data for future years. For the 2019 scenario, the initial conditions (IC) and boundary conditions (BC) for the CMAQ were derived from the 2016 seasonal average hemispheric CMAQ outputs, sourced from the CMAQ data warehouse.^42^ To ensure consistency between the base year and future simulations, we used seasonal scaling to interpolate the 2019 ICs/BCs based on trends observed in the outputs from global ensemble simulations conducted with the Community Earth System Model, version 2, coupled with the Whole Atmosphere Community Climate Model version 6 (CESM2/WACCM6), under the moderate Shared Socioeconomic Pathway scenario SSP2-4.5.^43^ Similar Interpolation approaches have been taken by Mousavinezhad et al.^44^ and Woody et al.,^45^ in the absence of ICs/BCs for a particular year. Moreover, employing nested modeling domains with a relatively large parent domain helped minimize the impact of boundary conditions on the innermost domain.

Biogenic emissions were estimated using MEGAN v3.1, which calculates biogenic compound fluxes from terrestrial ecosystems into the atmosphere, incorporating mechanistic algorithms and influenced by soil NO_*x*_ emissions.^46^ Meteorological inputs for MEGAN came from the WRF model, and leaf area index (LAI) values were updated by using MODIS satellite data (MCD15A2H; 45) to reflect interannual vegetation variability. For consistency, the same base year LAI and land use data were maintained across all scenarios.^47^ Emissions from other natural sources were calculated inline as detailed by Dajnak et al.^20^ All emissions were processed and converted to the CMAQ format using the Sparse Matrix Operator Kernel Emissions (SMOKE) system.^48^ The CMAQ-urban model assessed major UK road sources, segmenting the road network into 10 m sections with specific emissions and characteristics. Dispersion was modeled using a kernel approach from ADMS-Roads,^38^ with contributions aggregated onto a 20 m × 20 m grid near roads. Road types were categorized into open, typical, and street canyons, each with specific dispersion characteristics. To prevent double counting in the CMAQ model, road traffic pollutant concentrations derived from ADMS within each 2 km grid square were integrated, divided by the number of 20 m grid points, and subtracted from the CMAQ species total only at points covered by ADMS dispersion kernels.

While average concentrations are key for evaluating overall air quality, population-weighted average concentrations (PWAC) are instrumental in assessing exposure to air pollutants, a critical aspect of air quality management and public health policy. Research by ApSimon et al.^49^ has demonstrated that PWAC and population-weighted measures are less sensitive to model uncertainties and offer more stable indicators of changes under future emission scenarios. Hence, in this study, we calculated PWAC for each pollutant and scenario, by combining the annual average concentrations in each of the 8887 UK wards with their respective populations. More details regarding the PWAC calculations are provided in Section S4. These population figures were then used as weights to calculate a weighted average concentration for each local authority and country and for the UK.

## Results and Discussion

### Model Evaluation

A comprehensive evaluation of the WRF and CMAQ-urban models for the 2019 base year has been undertaken and is provided in detail in Section S3. In summary, the WRF model shows high accuracy in predicting temperature (*T*), wind speed (WS), and relative humidity (RH), with correlation coefficients (*r*) of 0.96, 0.80, and 079, respectively, accompanied by minimal biases below 10% for all these parameters. For air quality, the CMAQ-urban model exhibits robust performance for NO_2_, PM_10_, PM_2.5_, and O_3_ predictions from rural to kerbside locations with small biases and *r* values of 0.79, 0.66, 0.71, and 0.88 respectively. Additionally, the model’s performance in estimating PM_2.5_ components was also evaluated against monitoring sites across the UK (Figure S12). The model demonstrated good overall accuracy in predicting noncarbonaceous PM_2.5_ components at 16 rural background sites. Notably, it showed acceptable predictions for ammonium (NH_4_), nitrate (NO_3_), sulfate (SO_4_), and Sodium (NA) with slight underestimations, while Chloride (CL) was more significantly underestimated. The comparison of carbonaceous PM_2.5_ species, constrained to just two rural monitoring stations, reveals a close match for organic carbon (OC) and a slight overestimation for Elemental Carbon (EC).

### Emission Forecasts Between 2019 and 2050

Table S1 details emission changes within the European Union (EU) from 2019 to 2050, with neighboring UK countries projected to significantly reduce all key pollutants except NH_3_, which may increase by up to 48% due to stable or rising agricultural activities which comprises about 94% of the total EU NH_3_ emissions. The changes in pollutant emissions by 2050 for these countries range from 48 to −6% for NH_3_, from −23 to −76% for SO_2_, from −50 to −66% for NO_*x*_, from −25 to −67% for PM_2.5_/PM_10_, and from −10 to −27% for VOCs.

In the UK, as outlined in Table S3, emission forecasts for the BAU scenario between 2019 and 2040 show substantial reductions in emissions, with NO_*x*_ projected to decrease by 38% and SO_2_ by 47%, alongside more modest reductions for PM_2.5_ (18%), and PM_10_ (8%). In line with other EU nations, there is no significant reduction in NH_3_ emissions, with only a 1% increase projected. VOC emissions, while declining in most sectors, show less than a + 1% overall change due to increased solvent use (SNAP 6). The major contributor to NO_*x*_ reduction by 2030 is a 76% decrease in road emissions (SNAP 7), primarily due to lower emissions from new vehicles. An additional 11% reduction will occur by 2040. Further NO_*x*_ reductions are expected under the BNZP and WI scenarios, driven by low-carbon heating, faster EV adoption, and reduced vehicle VKM. The scale of reduction in the WI scenario is slightly lower, predominantly because of higher VKM and the slower uptake of zero-emission technology among larger heavy goods vehicles (HGVs), as hydrogen options are crowded out by battery technologies (Figure S1 and Table S2).

At the time of this study, NZ policies for buildings had not been implemented in the BAU scenario; therefore, no significant changes in NO_*x*_ emissions from combustion in domestic and commercial buildings (SNAP2) were projected for 2030 and 2040. However, the BNZP scenario anticipates considerable reductions in total NO_*x*_ emissions from this sector with projected decreases of 31% by 2030, 69% by 2040, and 98% by 2050. This highlights the marked impact of targeted policy interventions particularly in the SNAP2 sector on UK emission totals. Future building emissions are the same for BNZP and WI.

Buildings are the largest contributors to UK PM_2.5_ emissions in 2019, accounting for 33% of the total (Table S3). This is primarily due to combustion activities in residential and commercial buildings, with more than half of these emissions arising from wood burning for heating. The remaining PM_2.5_ emissions are from the use of gas, coal, and other fuels. Under the BNZP scenario, however, a substantial decrease in emissions is anticipated, with a predicted 10 and 54% further reduction in both PM_10_ and PM_2.5_ emissions from buildings in 2030 and 2040, respectively, compared to BAU, largely due to a shift toward low-carbon heating. For road transport emissions, an 18% decrease in PM_2.5_ emissions was predicted by 2030 under the BAU scenario, followed by a slight increase (4%) by 2040, mainly due to a predicted 35% increase in VKM by 2040. This slight increase is despite the significant growth of 33% in EVs (see Table S2), combined with our emissions estimates, which suggest that a like-for-like change from petrol or diesel vehicles to the equivalent EV would lower nonexhaust PM_10_ and PM_2.5_ emissions from light-duty vehicles by approximately 30% as well as removing exhaust emissions altogether. Notably, this finding challenges the concerns of Williams et al.^16^ about the potential rise in nonexhaust PM emissions from EVs. The nonexhaust decrease is largely due to a 64–95% reduction in brake wear from regenerative braking,^50^ which is partially offset by moderate rises in tire wear, road wear, and resuspension emissions, as EVs are generally heavier than equivalent combustion engine vehicles.^51−53^ The BNZP scenario forecasts greater reductions in both PM_10_ and PM_2.5_ emissions from road transport, with PM_10_ emissions projected to decrease by 12% in 2030 and 21% in 2040, and PM_2.5_ emissions by 12% in 2030 and 24% in 2040, compared to the BAU scenario. These reductions are primarily driven by the accelerated adoption of EVs and reduced VKM. PM emissions reductions in the WI scenario are similar to those in BNZP, though slightly lower, due to increased VKM (see Figure S1).

### Air Pollution Predictions in the UK Between 2019 and 2050

#### BAU Scenario

**Figure 2:**
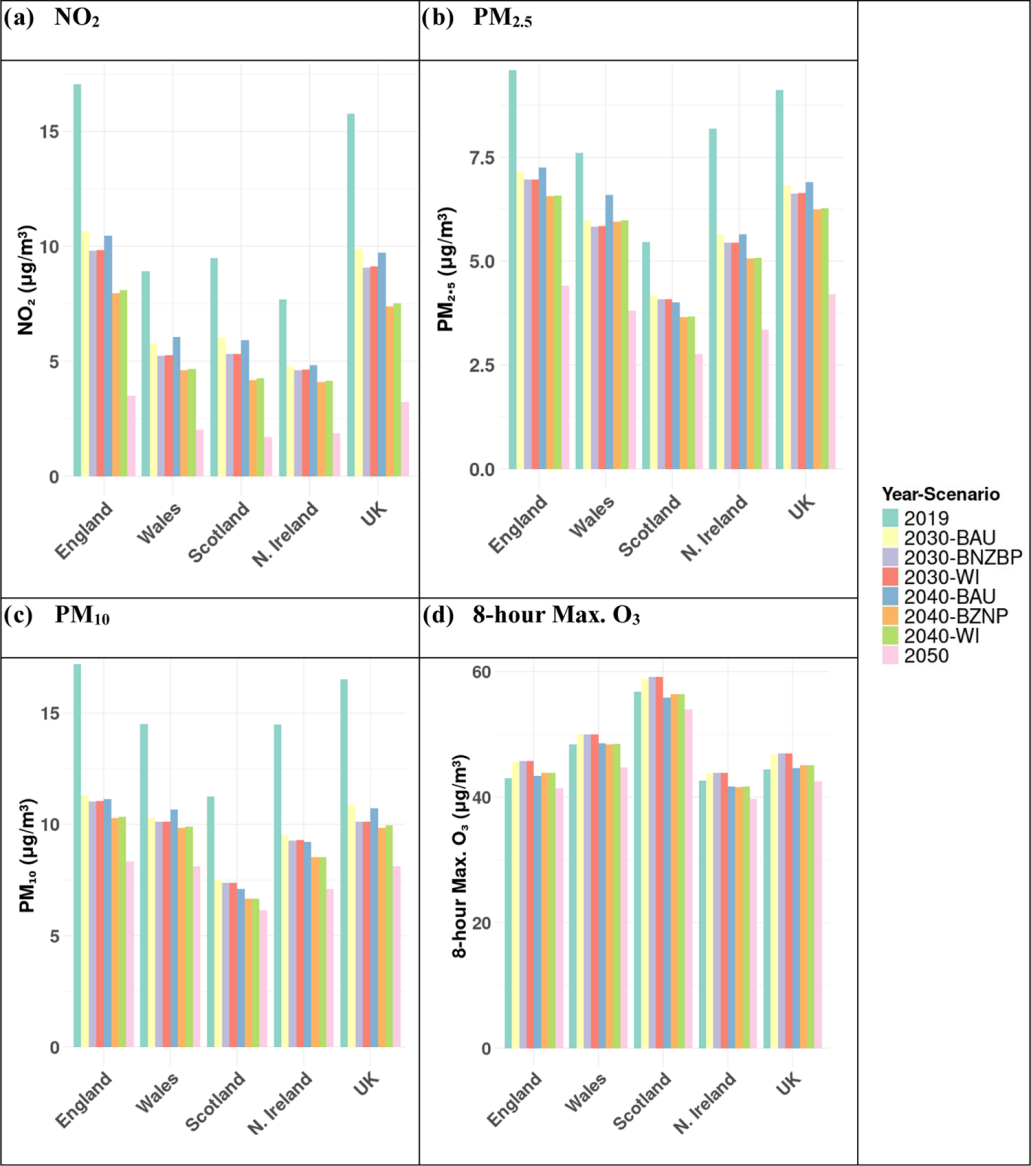
Population-weighted average concentration by country and Scenario for NO_2_ (a), PM_2.5_ (b), PM_10_ (c), and O_3_ (d). ,b.Concentration values are given in Table S7.

The UK model predictions for annual average NO_2_ in 2019 (Figure S13a) indicate that numerous locations, especially in major urban areas and near roads, exhibit NO_2_ concentrations exceeding 30 μg m^–3^. With the emission reduction strategies outlined in the BAU scenario, driven largely by a decrease in road emissions and improved vehicle technology with stricter emission standards, an overall improvement in NO_2_ concentrations is predicted in 2030BAU (Figure S13b). The NO_2_ PWAC for 2030BAU (Figure 2a) also suggests a notable reduction in population-weighted exposure, in the UK the concentration drops from 15.78 μg m^–3^ in 2019 to 9.88 μg m^–3^ in 2030BAU. However, this reduction is not uniform across the UK, with Wales, Scotland, and Northern Ireland showing smaller decreases, reflecting their lower population densities and 2019 concentrations. Transition from 2030BAU to 2040BAU (Figure S13c) reveals minimal changes across most areas, attributed to limited emission changes (Table S3). This is also reflected in the marginal changes of the NO_2_ PWAC values between the 2030BAU and 2040BAU scenarios, showing an overall slight decrease across the UK (Figure 2a).

The UK model forecasts for annual average PM_2.5_ in 2019 (Figure S14a) vary spatially across regions. In the north and west of England, Scotland, Wales, and Northern Ireland, most areas remained below 10 μg m^–3^. However, several locations, notably in major cities, have predicted PM_2.5_ concentrations above this threshold. In contrast, the 2030BAU scenario (Figure S14b) forecasts widespread reductions in PM_2.5_ throughout the UK where it drops from 9.13 to 6.82 μg m^–3^ (Figure 2b), with the most significant improvements, in previously polluted areas. This change is partly due to a decrease in local primary emissions, especially from the building and industrial combustion sectors (Table S3). More significantly, it is linked to reductions in secondary inorganic PM, reflecting the decline in NO_*x*_ emissions from road transport. This aligns with observed changes in PM_2.5_ components at monitoring sites (Figure S12), suggesting that the reduction from 2019 to 2030BAU is largely driven by a decrease in secondary inorganic aerosols (SIA—nitrate, sulfate, and ammonium), from 3.9 to 1.9 μg m^–3^. It is important to note that while SIA can originate both locally and from long-range transport, the overall reduction in PM_2.5_ also includes a 7% decrease in transboundary PM_2.5_. This transboundary reduction, accounting for sources outside the UK such as international shipping and European emissions, contributes to the overall decrease in PM_2.5_ concentrations, alongside local reductions in SIA as highlighted by ApSimon et al.^49^

Transitioning from the 2030BAU to 2040BAU scenario (Figure S14c), the observed trends in PM_2.5_ concentrations present a complex picture. While most regions across Eastern and Southeastern England, Scotland, and Northern Ireland exhibit a marginal overall decrease, slight increases are also observed in certain areas, particularly in coastal regions and parts of Wales, and along some major roads. The overall change is small mainly due to small emissions changes, with meteorological differences between 2 years becoming more influential. Aligned with these trends, the PWAC estimates for PM_2.5_ (Figure 2b) reveal a complex picture across the UK, with a marginal overall increase of 0.1 μg m^–3^.

The concentration maps for PM_10_ for 2019 and future scenarios are shown in Figure S15. The distribution patterns of PM_10_ and the changes in future scenarios closely resemble those observed for PM_2.5_. Notably, coastal regions show a distinct influence from sea salt on PM_10_ levels. While the factors affecting PM_2.5_ also impact PM_10_, the role of nonexhaust emissions from road transport is more pronounced for PM_10_. This is due to their larger contribution to PM_10_ compared to PM_2.5_. For instance, the increased traffic projected in the 2040BAU scenario (Figure S1), relative to 2030BAU, leads to an increase in PM_10_ concentrations along major roads (Figure S15g).

The 2019 model forecasts for annual average O_3_ (Figure S16a) reveal a varied spatial distribution across the UK, displaying an inverse correlation with NO_*x*_ emissions, particularly in urban areas where higher NO_*x*_ results in lower O_3_ due to titration effects.^54^ The year 2019 was characterized by warmer-than-average temperatures in the UK, especially during the winter months,^55^ with a number of heatwaves occurring in summer (Figures S17a–S20a). These elevated temperatures facilitated O_3_ formation, leading to episodic meteorology-driven increases in daily maximum O_3_ concentrations (Figures S17b–S20b). The 2030BAU scenario predicts an overall rise in O_3_, especially near major roads and in cities, as NO_*x*_ reductions decrease O_3_ titration (Figure S16b). The daily maximum O_3_ and temperature time series for 2030 show no similar high-temperature events to 2019, indicating a minimal meteorological impact on the projected O_3_ increases. A notable decrease in UK biogenic isoprene emissions supports this finding (Table S3). In the 2040BAU scenario (Figure S16c), the opposite observation is made. Despite the continued reduction in total NO_*x*_ emissions, there is an overall decrease in O_3_ concentrations across most parts of the UK, in contrast to the trends observed for 2030BAU. This shift in the O_3_ trend for 2040 can be attributed to several interplaying factors. First, the reduction in local and regional anthropogenic precursor emissions plays a significant role. Concurrently, there is an observed increase in biogenic VOCs (BVOCs) emissions under warmer conditions, with an increase in UK isoprene emissions (Table S3). These changes in isoprene levels, in conjunction with the evolving anthropogenic emissions, alter the NO_*x*_ to VOCs ratio, a critical factor in O_3_ formation. Additionally, changes in the atmospheric oxidation capacity, marked by variations in the levels of OH and other oxidants, play a pivotal role in determining the rate of O_3_ formation and degradation. At the same time, long-range transport of O_3_ and its precursors interplays with these local changes.

#### NZ Policies

**Figure 3:**
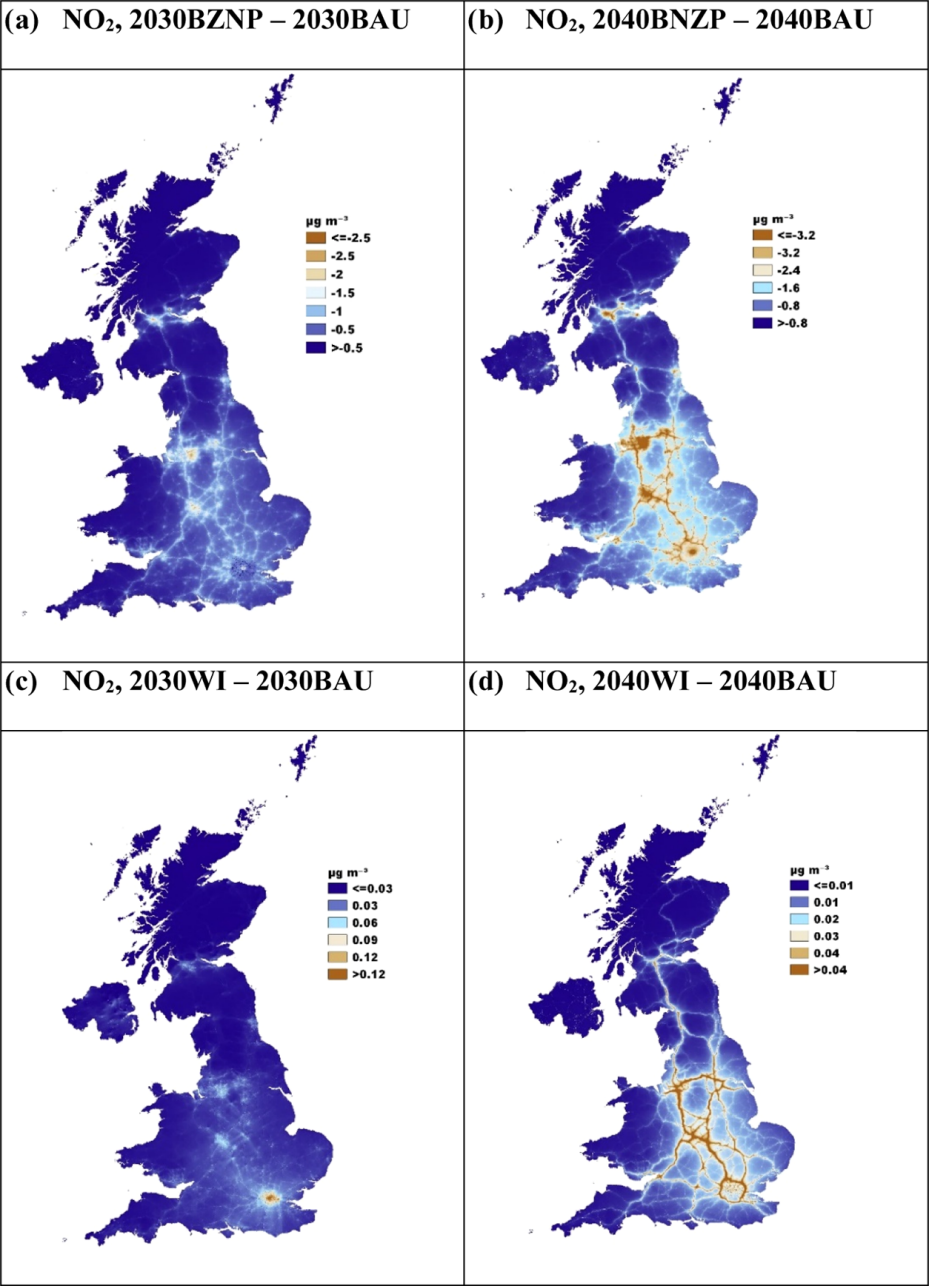
CMAQ-urban UK difference maps for NO_2_ concentrations: (a) 2030BNZP and 2030BAU, (b) 2040BNZP and 2040BAU, (c) 2030WI and 2030BAU, and (d) 2040WI and 2040BAU.

The BNZP scenario for both 2030 (Figure 3a) and 2040 (Figure 3b) has more significant declines in NO_2_ concentrations compared to BAU, especially along major roads and in cities. The improvements are more pronounced by 2040, as the cumulative effects of reductions in emissions from both road traffic and buildings become more evident. NO_2_ source apportionment results comparing BNZP with BAU in 2030 (Figure S22a) support this, showing a marked decrease in the contribution from buildings and road transport to the UK’s total annual average NO_2_ concentration, by 24 and 20% respectively. The size of these reductions is larger in 2040, with contributions from buildings and road transport decreasing by 67 and 74%, respectively. Correspondingly, the PWAC figures (Figure 2a) for BNZP and WI show that average UK exposure to NO_2_ decreases by 0.8 μg m^–3^ in 2030 and 2.4 μg m^–3^ in 2040 compared with BAU. The spatial variations in PWAC changes across local authorities in Figures 6a and S21a further highlight the disparities in policy impact and are attributable to factors such as 2019 pollution concentrations, road traffic density, the rate of EV adoption, and the extent of low-carbon heating adaptation in buildings. Urban areas are poised to benefit markedly from initiatives promoting electrification and VKM reduction. These findings align with those of Williams et al.^16^ and Zhong et al.,^19^ who, despite using different emission assumptions and policy frameworks, also predicted notable NO_2_ reductions through vehicle electrification. Moreover, the adoption of energy efficiency measures and the deployment of heat pumps, specifically targeting fuel-poor homes, social housing, and tenure, and implemented across different regions and housing types, are responsible for the observed spatial variation in the impacts of NZ policies. For London, due to the city’s emissions policies, there were substantial reductions in NO_2_ for the 2030BAU scenario (Figure S13b) and so the 2030BNZP scenario (Figure 3a) resulted in smaller reductions, compared to other urban areas. While the NO_2_ concentrations in the WI scenario are broadly similar to those in the BNZP for both 2030 (Figure 3c) and 2040 (Figure 3d), there is a slight increase in NO_2_ concentrations along major roadways in the WI scenario. This is consistent with marginally higher emissions projected under the WI scenario, which is due to a slower conversion of heavy vehicles to zero tailpipe technologies and, hence, more diesel vehicles. Looking ahead to 2050 (Figure S13d), under the BNZP scenario, plus reductions to other sources in the UK and Europe, we predicted that most areas will experience a dramatic decrease in NO_2_ concentrations, with a few exceptions in London in the vicinity of Heathrow Airport.

**Figure 4:**
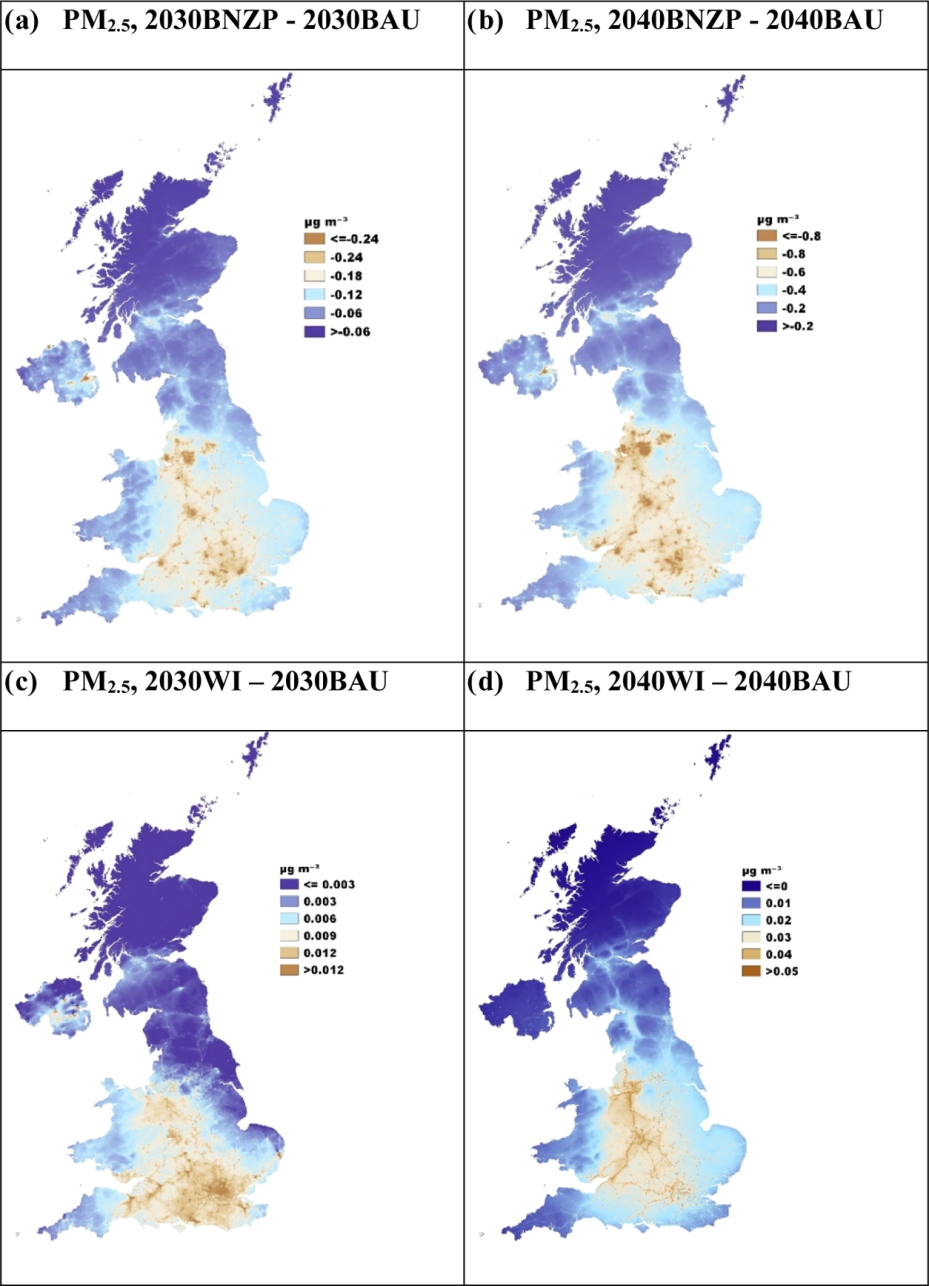
CMAQ-urban UK difference maps for PM2.5 concentrations: (a) 2030BNZP and 2030BAU, (b) 2040BNZP and 2040BAU, (c) 2030WI and 2030BAU, and (d) 2040WI and 2040BAU.

Similarly, the BNZP scenarios for both 2030 and 2040 (Figure 4a,b) resulted in additional PM_2.5_ reductions when compared to those of the respective BAU scenarios. These improvements are most pronounced in areas close to roads and urban centers, a result of NZ emission control strategies targeting vehicles and buildings. The observed change is supported by PM_2.5_ source apportionment results (Figure S22b), which show decreases in contributions to the UK’s total annual average PM_2.5_ concentration in the BNZP scenario compared to BAU. Specifically, there is a decrease of 13% from buildings and 12% from road transport for 2030, and by 55% from buildings and 41% from road transport for 2040. Notably, the absolute reduction from buildings is on average twice as large as that from road transport. Similarly, the PWAC values (Figure 2b) for BNZP and WI show further average decreases compared to BAU across the UK, of 0.2 μg m^–3^ in 2030 and 0.6 μg m^–3^ in 2040 but with a range for each UK local authority, as illustrated in Figure 6b. The WI scenarios for 2030 and 2040 (Figure 4c,d), however, show a noticeable yet marginal increase in PM_2.5_ concentrations along major roadways compared with BNZP, for the reasons discussed previously in the emissions sections. By 2050 (Figure S14d), the BNZP scenario projects a significant reduction in PM_2.5_ across the UK, moving toward net zero targets. The expected PM_2.5_ PWAC for the UK under the 2050BNZP scenario indicates a dramatic decline to 4.2 μg m^–3^, a significant reduction from the 2019 level of 9.1 μg m^–3^. As for PM_10_, similar to NO_2_ and PM_2.5_, 2030BNZP (Figure S15c) and 2040BNZP (Figure S15e) scenarios predict more substantial reductions compared to the respective BAU scenarios, especially near urban centers and roads, with PM_10_ concentrations expected to decrease from 10.9 μg m^–3^ in the 2030BAU scenario to 9.9 μg m^–3^ in the 2040BNZP scenario (Figure 2).

**Figure 5:**
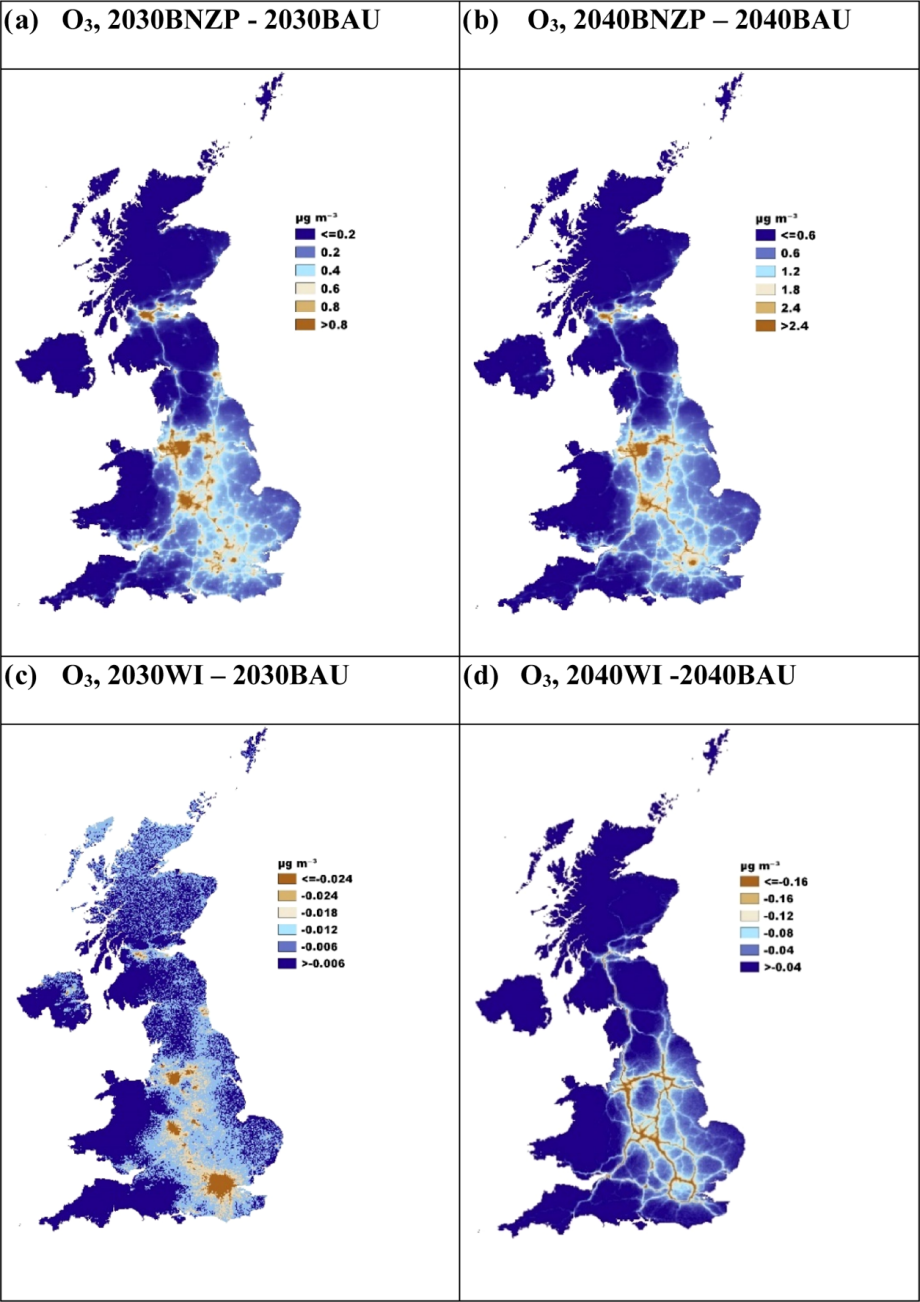
CMAQ-urban UK difference maps for the O_3_ concentrations: (a) 2030BNZP and 2030BAU, (b) 2040BNZP and 2040BAU, (c) 2030WI and 2030BAU, and (d) 2040WI and 2040BAU.

**Figure 6:**
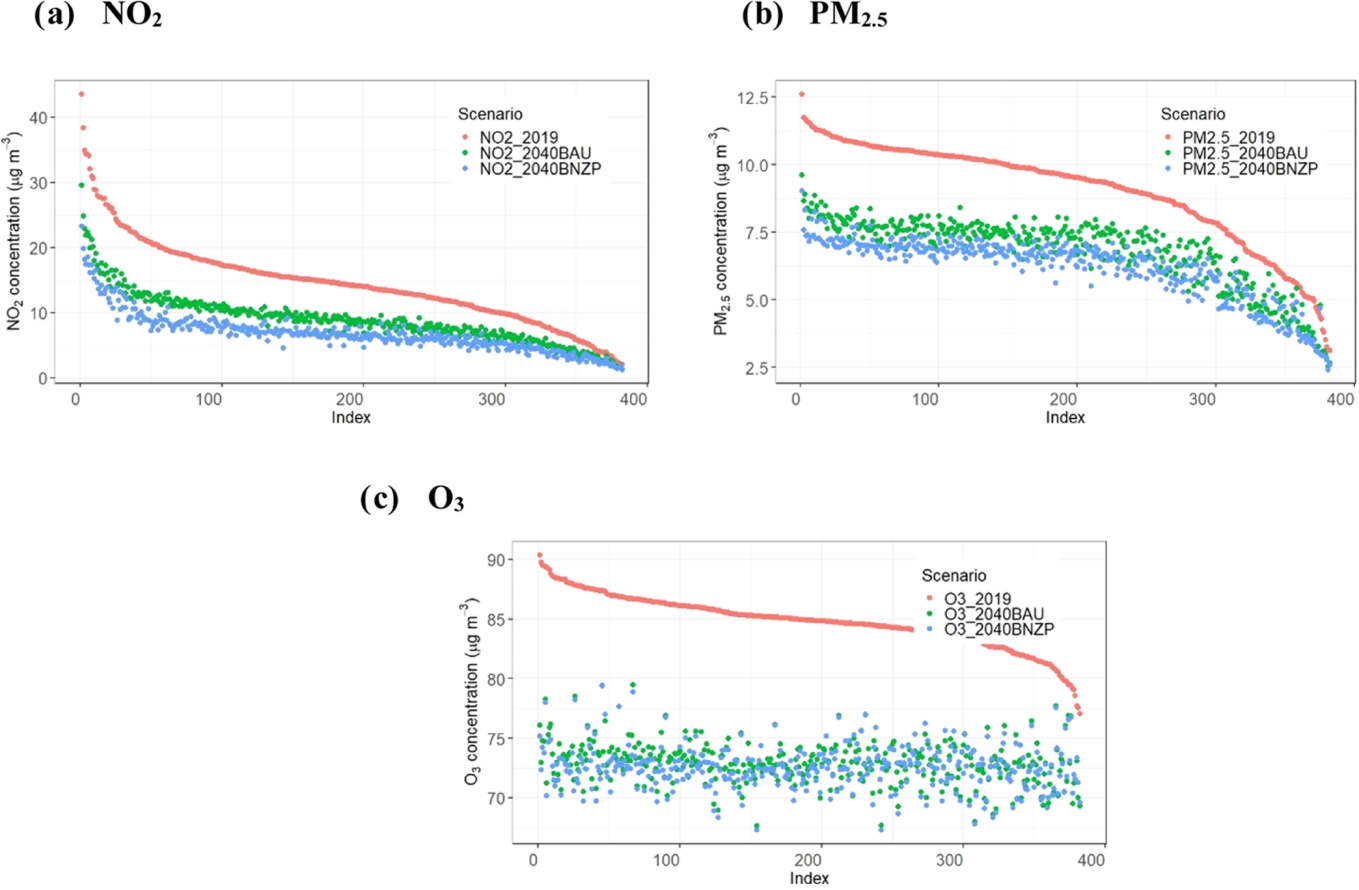
Population-weighted average concentrations (μg m^–3^) for NO_2_ (a), PM_2.5_ (b), and O_3_ (c) for every local authority in the UK (*n* = 382) – 2019, 2040BAU and 2040BNZP. Note that concentrations for all scenarios are in descending order based on the observed concentrations in the 2019 scenario.

For O_3_, the BNZP (Figure 5a,b) and WI (Figure 5c,d) predict an increase in O_3_ specially in cities and along roads compared to the BAU scenario, reflecting further reductions in NO_*x*_. By 2050 (Figure S16d), a further decline in the level of the O_3_ concentrations is predicted, predominantly driven by the continued reduction in local and regional anthropogenic emissions of precursors, outweighing the increase in the level of BVOCs. These patterns are also reflected in the UK’s PWAC estimates for 8 h maximum O_3_ (Figure 2), which show an increase from 44.4 μg m^–3^ in 2019 to 47.0 μg m^–3^ in 2030, before decreasing to 42.5 μg m^–3^ by 2050. Similar trends are forecast for each country within the UK. Figure 6c shows the 8 h maximum O_3_ PWAC for each local authority in the UK, comparing baseline 2019 with the projected 2040 under both BAU and BNZP scenarios. The results reveal a notable spatial heterogeneity in O_3_ concentrations across local authorities in 2040. While some areas exhibit increases in PWAC, others show reductions, reflecting the complex and nonlinear response of ozone to changes in precursor emissions. This variability may be attributed to several factors, including regional differences in precursor emissions (e.g., NO_*x*_ and VOCs), local meteorology, and long-range pollutant transport. These findings also highlight the intricate balance between the benefits of emission reductions (particularly NO_*x*_) and their unintended consequences for ozone concentrations. The projected increases in some areas suggest potential trade-offs, where reductions in NO_*x*_, which acts as an ozone scavenger in urban areas, could paradoxically lead to higher ozone levels, particularly in regions dominated by VOC-limited regimes. These observations emphasize the importance of adaptive and regionally tailored air quality management strategies that account for these complex dynamics. Future investigations should focus on the underlying drivers of the observed variations and explore mitigation strategies that consider the multipollutant nature of air quality management. A holistic approach will be crucial to optimizing the cobenefits of climate policies while minimizing adverse impacts on O_3_ pollution.

Since our analysis of NZ policy highlighted the importance of the switch to low-carbon heating in buildings, we assessed the impact of NZ policies on indoor air pollution exposure, examining changes in outdoor air quality, modifications in home characteristics, and adjustments in indoor sources. Details of this analysis are provided in Section S5. Indoor pollution may arise from direct emissions (e.g., from cooking and heating), resuspension, or the ingress of outdoor air. The exchange of air pollution between indoor and outdoor environments is chiefly regulated by home ventilation, which is influenced by the characteristics of the home and the climatic conditions both inside and outside. Figure S26 shows the relative change of indoor exposure to PM_2.5_ and NO_2_ under different scenarios for homes in 2019 and 2040, compared with their outdoor concentrations measured in 2019. In summary, the NZ policies on road transport and building heating have significant impacts on improving outdoor air quality, thereby reducing exposure to indoor air pollutants originating from outdoor sources. Transitioning from gas to electric cooking also reduces exposure to indoor pollutants, particularly NO_2_. However, home insulation may yield both positive and negative effects on exposure, depending on home ventilation settings and the frequency and emission intensity of indoor sources of air pollutants. Owing to a lack of measurements, the study has not encompassed the impact of eliminating other indoor fossil fuel burnings such as domestic wood burning. Furthermore, due to the study scope, we have only examined a case study in London homes and two indoor air pollutants, PM_2.5_ and NO_2_. Despite these limitations, this case study offers insights into the investigation of the health impacts of indoor air pollution in future studies of NZ.

We also acknowledge several sources of uncertainty that affect our findings. First, even though the air quality and meteorological models exhibit overall good performance in the 2019 base year, as evidenced by high correlation coefficients and acceptable bias ranges, there remains inherent uncertainty. This is due to their dependence on accurately capturing complex atmospheric chemistry, emissions data, and meteorological conditions. While we account for changes in meteorology by using forecasted meteorological fields for future year scenarios, we also acknowledge that meteorology plays an important role in shaping air pollution concentrations. In recognition of the influence that meteorology will play further into the future, we have limited our 2019 base year comparisons to the 2030BAU scenario. However, to further explore the role of meteorology in predicting 2030 air pollution, we conducted a sensitivity analysis by running the CMAQ model using 2030BAU emissions and 2019 meteorological fields. The results showed that while the differences in annual mean pollutant concentrations between 2030BAU and 2019 using different meteorological fields were large, the difference between air pollution predictions using 2030BAU emissions and both 2030 and 2019 meteorology was relatively small. Specifically, PM_10_ differed by 0.6 μg/m^3^, PM_2.5_ by 0.3 μg/m^3^, NO_2_ by 0.2 μg/m^3^, and O_3_ by 0.6 μg/m^3^, supporting the conclusion that in the near term air pollution changes are mainly influenced by emissions reductions. Although 2019 experienced higher-than-average summer temperatures, the similarity in air quality outcomes for 2030 may reflect this. Moreover, in a separate study using 2018 meteorological fields with a 2030BNZP road transport-only scenario and the same modeling methods, we observed comparable results.^20^ This also agrees with evidence from previous studies^20,39^ suggesting that over shorter time horizons (e.g., 10 years), changes in air pollution are primarily driven by shifts in anthropogenic emissions rather than meteorological variability. Second, the assumptions underpinning emissions forecasts and scenario projections, such as the adoption rates of electric vehicles and advancements in renewable energy technologies, add another layer of uncertainty. For instance, despite the increased electricity demand from the use of electric vehicles and in building heating, future projections of electricity generation from the sixth Carbon Budget show a decline in the use of fossil fuels for this purpose. Additionally, from our source apportionment model results, there is a less than 1% contribution to UK annual average NO_2_ and PM_2.5_ concentrations, in 2019, from electricity-generating sources, justifying the exclusion of this impact in the overall analysis. It is important to note however that these are reliant upon continued development of UK offshore electricity generation. These factors, coupled with potential deviations in policy implementation and behavioral changes, influence the accuracy of our predictions. Future research must focus on refining these models and assumptions, as well as incorporating broader variables and up-to-date data, to enhance the reliability of such studies in guiding effective NZ policies and air quality management strategies. The comparison between BAU and BNZP/WI also assumes that future BAU target emissions reductions are achieved, which from experience over previous decades may not be the case.

This study contributes to international evidence of the effects of NZ and is widely applicable to other countries that have similar aims. While each country devises its strategy and policy framework toward achieving NZ emissions based on a variety of factors, including existing energy infrastructure, economic conditions, political landscapes, and societal readiness, it is crucial to underscore that combating climate change is inherently a global endeavor. Climate change transcends national boundaries, making it imperative to examine the different aspects and assess the cobenefits or trade-offs of each nation’s progress toward NZ. The diversity in energy sources, technological advancements, financial capabilities, and public acceptance plays a significant role in shaping each country’s journey to NZ. For example, Norway’s progress in housing electrification and high EV uptake serves as a successful model for reducing emissions in the residential sector and transportation. Investigating air quality cobenefits, Grythe et al.^56^ observed a significant reduction in light vehicle CO_2_ emissions by 22% since 2009 in Norway, primarily due to the rising adoption of EVs and biofuel integration. Moreover, this transition halved NO_*x*_ emissions from their peak in 2014, mirroring our findings in the UK that underscore the benefits NZ scenarios would offer in terms of emission reduction and air quality improvement. We also explored the spatial variations in the impact of NZ policy on air quality in the UK. Similarly, Mousavinezhad et al.^44^ examined the United States, uncovering the nuanced and region-specific impacts of vehicle electrification on air quality. Although reductions in PM_2.5_ were evident, the study also identified potential unintended consequences, such as an increase in SOA levels in places like Los Angeles, highlighting the necessity for a comprehensive monitoring system to identify and mitigate any adverse effects of electrification. In contrast, in the UK, the CCC anticipates that offshore wind energy production will be sufficient to meet the rising electricity demand. It is imperative to not only rely on these forecasts but also ensure their realization through vigilant planning and policy implementation. Together, these international case studies, alongside our UK-focused analysis, emphasize the critical need for holistic policy frameworks that simultaneously tackle air pollution and carbon emissions. By drawing on these insights, we can better inform and guide global efforts to achieve ambitious environmental targets, illustrating the interconnected nature of these challenges and the shared benefits of concerted action.

## Supporting Information Available

Section S1 includes the European emission changes between the base year and future years by country, assumptions of BAU and NZ emissions scenarios, and total UK emissions under each scenario by sector. Section S2 details the configurations of the air quality and meteorology models. Section S3 presents the performance evaluation results of the meteorology and air quality models. Additional modeling results and maps are provided in Section S4, while Section S5 covers the supplementary indoor air quality modeling. (PDF)
