# Supplementary material for: What Impact Does Net Zero Action on Road Transport and Building Heating Have on Exposure to UK Air Pollution?
Source: Environ Sci Technol. 2025 Jan 10;59(2):1274–86. doi: 10.1021/acs.est.4c05601 (PMC11755711; doi:10.1021/acs.est.4c05601)
Supplement: Supplementary file 1 — es4c05601_si_001.pdf [file es4c05601_si_001.pdf]

SUPPORTING INFORMATION

What impact does Net Zero action on road transport and building heating have on exposure to UK air pollution?

Nosha Assareh [a,b*], Andrew Beddows [a,b], Gregor Stewart [a,b], Mike Holland [c], Daniela Fecht [d],

Heather Walton [a,b,e], Dimitris Evangelopoulos [a,b], Dylan Wood [a.,b], Tuan Vu [a,b], David Dajnak [a,b],

Christian Brand[f], and Sean David Beevers [a,b,e]

[a] Environmental Research Group, School of Public Health, Imperial College London, Sir Michael Uren Biomedical Engineering Hub, White City Campus, 80 Wood Lane, W12 0BZ, London, United Kingdom.

[b] MRC Centre for Environment and Health, School of Public Health, Imperial College London, Sir Michael Uren Biomedical Engineering Hub, White City Campus, 80 Wood Lane, W12 0BZ, London, United Kingdom.

[c] Ecometrics Research and Consulting, RG8 7PW, Reading, United Kingdom.

[d] School of Public Health, Faculty of Medicine, Imperial College London, White City Campus, 80 Wood Lane, W12 0BZ, London, United Kingdom.

[e] NIHR HPRU in Environmental Exposures and Health, School of Public Health, Imperial College London, Sir Michael Uren Biomedical Engineering Hub, White City Campus, 80 Wood Lane, W12 0BZ, London, United Kingdom.

[f] Transport Studies Unit, University of Oxford, South Parks Road, Oxford, OX1 3QY, United Kingdom.

[*] Email: n.assareh@imperial.ac.uk

Summary: 49 pages, 26 figures, 8 tables

Section S1 Emissions forecasts and methodology

Non-UK emissions

European emissions of NO_X, CO, PM_{10}, $PM_{2.5}$, SO_2, HCl, VOCs and NH_3 were acquired from the European Monitoring and Evaluation Programme (EMEP) Centre on Emission Inventories and Projections (CEIP) for 2019 and summarized as a set of 50km grids. The emissions were classified into 11 Selected Nomenclature for Air Pollution (SNAP) source types and the anthropogenic emissions further processed into hourly gridded chemical species using methods developed in the US-EU 'Air Quality Modelling Evaluation International Initiative' (AQMEII) project (1). Future European emissions projections for each nation state were taken from the European Commission's Second Clean Air Outlook v2021 (2), providing total emissions for all pollutants, by snap sector, for each nation from now until 2050.

Table S1 European emissions % changes between 2019 and 2030,2040, and 2050 by country

Country	NH_3			SO_2			NO_x			$PM_{2.5}$			VOC		
	2030	2040	2050	2030	2040	2050	2030	2040	2050	2030	2040	2050	2030	2040	2050
Albania	20.6	27.3	38.5	-1.7	-6.6	17.5	-16.1	-30.0	-27.6	-27.3	-39.9	-39.3	-29.6	-40.3	-41.2
Armenia	16.0	1.3	0.0	39.9	57.2	81.8	5.7	17.8	40.4	-3.4	2.8	12.5	-14.5	-1.4	11.5
Austria	2.8	22.7	32.4	-24.9	-30.9	-32.3	-54.8	-61.0	-61.3	-36.6	-41.8	-42.7	-22.0	-28.0	-29.9
Azerbaijan	12.8	8.7	7.4	6.4	19.9	36.0	32.3	56.0	79.3	28.2	47.2	64.1	29.8	35.7	44.3
Belarus	9.8	-3.1	-5.2	3.9	5.7	-4.4	-5.9	2.2	4.2	8.6	12.1	10.5	-15.0	-10.8	-8.6
**Belgium**	**-0.7**	**31.9**	**47.8**	**-23.2**	**-33.5**	**-34.0**	**-39.6**	**-53.6**	**-49.6**	**-30.3**	**-49.4**	**-55.2**	**-10.8**	**-18.0**	**-17.2**
Bosnia-H	14.6	2.3	0.4	-60.6	-66.7	-65.2	-34.1	-51.2	-52.9	-14.6	-26.2	-32.4	-16.3	-28.9	-36.2
Bulgaria	-0.4	-1.4	1.4	-42.5	-65.0	-72.3	-31.4	-44.3	-53.8	-57.0	-64.5	-71.2	-33.3	-38.9	-40.5

Croatia	1.7	6.5	11.6	-41.6	-55.3	-54.9	-44.6	-50.6	-52.6	-56.5	-61.4	-62.7	-21.6	-22.8	-23.6
Cyprus	1.8	-11.5	-10.1	-62.6	-49.9	-51.9	-40.6	-44.5	-44.0	-27.7	-26.8	-22.4	-20.7	-25.2	-18.8
Czech Republic	-7.8	-2.5	-4.7	-37.4	-71.9	-76.7	-31.3	-55.6	-58.3	-49.3	-63.3	-66.4	-21.8	-23.0	-21.1
**Denmark**	**-1.3**	**2.7**	**3.2**	**-30.3**	**-32.9**	**-31.2**	**-40.2**	**-53.3**	**-55.5**	**-51.8**	**-64.8**	**-67.0**	**-21.3**	**-26.6**	**-27.5**
Estonia	2.6	2.9	6.5	-26.2	-68.7	-76.6	-29.7	-56.5	-64.5	-52.1	-67.6	-73.9	-21.0	-19.7	-14.9
Finland	2.2	-3.4	-4.4	-32.6	-51.3	-52.6	-29.7	-48.2	-47.7	-29.0	-38.7	-38.7	-19.9	-25.9	-26.2
**France**	**-2.3**	**21.2**	**30.1**	**-37.9**	**-42.0**	**-44.1**	**-50.3**	**-62.4**	**-64.6**	**-47.1**	**-58.9**	**-59.2**	**-20.7**	**-25.7**	**-25.7**
Georgia	12.3	-11.5	-14.0	18.9	20.9	29.9	22.4	38.5	56.0	4.2	8.8	17.2	12.0	26.9	34.8
**Germany**	**-7.1**	**-2.2**	**-1.7**	**-38.7**	**-54.9**	**-62.7**	**-44.8**	**-61.7**	**-65.6**	**-19.5**	**-32.4**	**-37.5**	**-9.7**	**-14.2**	**-16.6**
Greece	-2.0	-11.9	-14.8	-55.9	-55.0	-73.7	-47.6	-56.4	-61.6	-43.5	-53.3	-57.6	-28.3	-33.6	-34.2
Hungry	-9.1	19.1	16.0	-56.2	-66.7	-63.9	-39.9	-51.2	-52.4	-58.2	-71.0	-76.5	-28.4	-34.0	-35.2
Iceland	-0.8	8.3	9.9	6.9	7.5	1.2	-5.1	-20.2	-36.7	-1.4	-11.2	-24.7	-2.1	-6.8	-13.5
**Ireland**	**5.5**	**-5.0**	**-6.0**	**-49.1**	**-74.6**	**-75.6**	**-36.7**	**-50.9**	**-52.0**	**-40.3**	**-60.7**	**-66.0**	**-12.8**	**-17.6**	**-17.4**
Italy	-2.8	2.2	5.9	-23.4	-35.1	-39.2	-45.6	-55.4	-58.0	-49.3	-66.3	-69.5	-18.3	-24.5	-24.6
Latvia	0.4	3.4	3.4	-11.5	-14.4	-15.7	-28.0	-51.0	-55.3	-51.0	-63.6	-72.2	-16.7	-21.2	-26.5
Lithuania	3.4	1.6	0.0	-24.8	-16.3	-20.7	-35.0	-49.0	-55.5	-64.4	-72.4	-76.9	-30.2	-32.4	-35.0
**Luxembourg**	**0.8**	**-3.2**	**0.2**	**-21.0**	**-25.1**	**-23.4**	**-60.4**	**-62.5**	**-61.8**	**-15.1**	**-38.0**	**-35.5**	**-13.8**	**-13.3**	**-9.9**
Malta	-4.4	7.6	11.8	-46.1	-49.5	-47.7	-39.6	-48.1	-48.8	-25.1	-29.1	-30.1	-12.4	-14.0	-15.6
Moldova	3.3	-7.7	-2.8	3.2	-5.8	-17.1	-16.5	-15.1	-15.6	-8.4	-8.0	-9.4	-26.1	-26.2	-27.7
Montenegro	-11.9	-5.1	-4.6	-87.6	-90.9	-92.0	-43.2	-63.9	-68.5	-25.3	-35.6	-44.4	-23.2	-33.8	-43.5
**Netherlands**	**-5.1**	**-0.2**	**2.8**	**-13.2**	**-28.7**	**-29.7**	**-38.9**	**-50.5**	**-53.4**	**-16.2**	**-23.4**	**-25.2**	**-7.6**	**-11.4**	**-12.3**
North Macedonia	-3.3	16.9	27.8	-41.3	-43.0	-39.8	-27.3	-33.9	-36.4	-11.3	-23.5	-27.4	-13.8	-30.4	-37.7
Norway	7.0	10.7	15.7	8.8	22.9	34.5	-25.7	-27.4	-25.9	-27.4	-36.8	12.2	-5.0	-5.5	2.3
Poland	6.9	3.4	3.8	-52.9	-70.6	-78.1	-35.0	-49.5	-55.2	-51.8	-65.7	-69.7	-29.2	-36.4	-37.7
Portugal	1.5	-4.0	-3.9	-25.7	-31.2	-35.2	-38.0	-49.8	-52.8	-37.2	-40.8	-44.3	-19.7	-21.5	-22.8
Romania	-4.3	7.9	9.7	-43.5	-58.3	-61.6	-33.1	-43.5	-45.9	-64.9	-68.4	-72.7	-42.7	-45.3	-47.1
Russia	5.9	-22.8	-22.7	-2.3	4.1	3.1	-12.7	-14.4	-31.6	-3.2	-3.5	-4.8	-8.2	-13.5	-17.0
Serbia	-20.3	-6.4	-6.7	-52.2	-59.1	-59.4	-23.2	-35.6	-47.5	-15.2	-37.2	-44.6	-20.5	-35.5	-43.0
Slovakia	-6.3	-6.1	-8.4	-53.8	-57.5	-59.3	-31.4	-43.7	-45.3	-43.7	-49.4	-54.2	-14.4	-16.2	-13.8
Slovenia	-4.1	1.5	2.7	-37.5	-77.2	-78.2	-43.1	-71.7	-74.1	-49.7	-69.5	-76.8	-20.5	-30.5	-33.9
Spain	0.3	1.1	6.9	-56.7	-55.7	-55.0	-45.7	-59.0	-61.3	-51.4	-59.5	-59.6	-12.0	-12.7	-12.6
Sweden	-1.8	-0.1	-0.6	-12.5	-27.2	-27.3	-48.1	-58.0	-57.7	-22.7	-24.3	-23.4	-9.1	-11.7	-10.6

Switzerland	-0.4	42.0	66.8	4.7	23.9	-17.1	-30.6	-38.2	-45.5	-0.9	5.1	0.0	-3.3	-2.5	-3.9
Turkey	17.4	5.9	7.4	-23.8	-37.2	-45.8	-11.5	-8.0	-9.2	0.2	1.3	3.9	-5.4	-6.6	-6.2
Ukraine	4.1	27.3	38.5	-15.4	10.7	26.0	7.8	23.1	31.4	17.7	35.3	41.2	-11.2	-8.9	-6.7

Note: countries geographically proximate to the UK are highlighted in bold font

UK Business As Usual (BAU) scenarios in 2019, 2030 and 2040

Anthropogenic emissions for the UK in 2019, for NO_X, CO, PM_{10}, $PM_{2.5}$, SO_2, HCl, VOCs and NH_3 were taken from the National Atmospheric Emissions Inventory (3). Non-road UK emissions were separated into SNAP sectors and processed into hourly 2km gridded species. The UK emissions forecasts between 2019 and 2030/2040 were taken from Department for Environment Food & Rural Affairs's (DEFRA) business as usual (BAU) scenario (NAEI v2020 year 2019, 2030 and 2040). DEFRA's forecast was based upon existing environmental policies (e.g. the Industrial Emissions Directive, Euro standards for vehicles) and energy forecasts from the Department for Business, Energy and Industrial Strategy (BEIS) (BEIS, 2019) but did not reflect measures under development for the UK's Clean Air Strategy.

In London, 2019 was based upon the London Atmospheric Emissions Inventory (5). London's 2030 BAU was based upon commitments made in the London Environment Strategy (6) and represent the current best emissions estimate in London in 2030. London's 2040 BAU was based upon the current best estimate of road transport emissions in London in 2040 and UK trends for non-road transport sources using NAEI v2020 projection between 2030 and 2040. As an alternative (net zero) scenario, the Climate Change Committee's (CCC) Balanced Net Zero Pathway (BNZP) scenario (7) was applied to London's road transport and residential, commercial and public buildings following the method described below. Note that in the case of cooking, a source that is missing from UK emissions altogether, cooking emissions in London was kept constant post 2030 due to a lack of information.

Emissions factors for UK road emissions

Road transport emissions of non-exhaust PM were calculated using data derived from a recent measurement campaign at Marylebone Road in London (8). Hybrid, plug-in hybrid and electric light-duty vehicles had further scaling factors applied to account for the effects of regenerative braking and increased vehicle weight, as simulated over the Worldwide Harmonised Light Vehicle Test Procedure (WLTP), Transport for London (TfL) drive cycle for outside London, and London respectively (9).

Exhaust PM emissions were calculated using factors from COPERT v5.4 (10), while new emissions curves derived from remote sensing data were used to calculate NO_x and primary NO_2 where sufficient measurement data were available; COPERT v5.4 functions were assumed for vehicle classes where such data were not available, including for all motorcycles and coaches.

Road Transport BNZP and WI Net Zero Scenarios

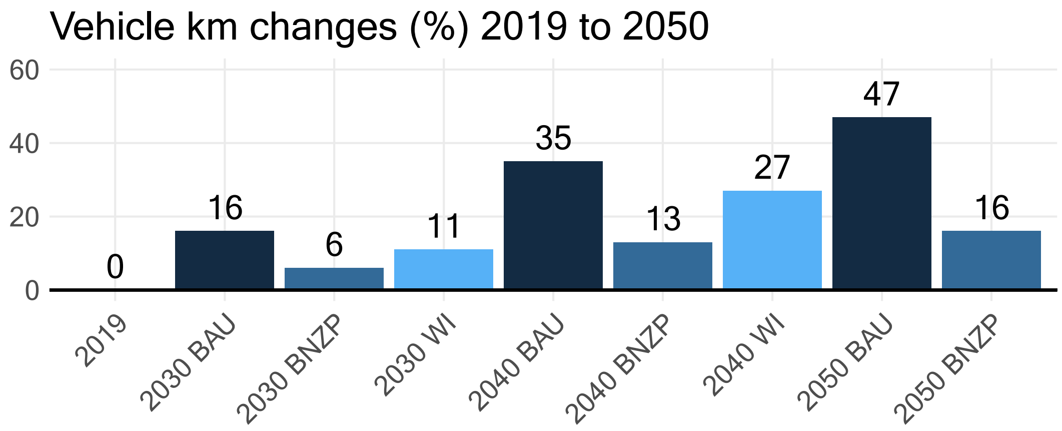

Figure S1 Change (%) in vehicle kilometers (VKM) travelled, between 2019 to 2050 for BAU, BNZP and WI scenarios

Table S2 Change (%) electrical vehicle (EV) and H2/Fuel cell vehicles (H2FC) by vehicle type between 2019 to 2050 for BAU, BNZP and WI scenarios.

	Car	LGV	Bus	Rigid	Artic

Year	Scenario	EV	EV	EV	H2FC	EV	H2FC	EV	H2FC
2019	BAU	0.5%	0.2%	0.0%	0.0%	0.0%	0.0%	0.0%	0.0%
2030	BAU	17.8%	4.0%	27.8%	0.0%	0.0%	0.0%	0.0%	0.0%
	BNZP	40.3%	42.1%	8.3%	9.0%	1.8%	0.8%	4.0%	1.1%
	WI	43.2%	42.2%	11.1%	0.0%	2.9%	0.0%	5.7%	0.0%
2040	BAU	33.4%	12.4%	46.2%	0.0%	0.0%	0.0%	0.0%	0.0%
	BNZP	88.8%	88.3%	25.5%	31.8%	26.6%	32.8%	37.3%	47.8%
	WI	90.2%	88.3%	48.1%	0.0%	58.0%	0.0%	61.5%	0.0%
2050	BNZP	100.0%	100.0%	43.7%	48.7%	55.6%	39.9%	59.3%	39.7%

Note: LGV: Light good H2FC: Hydrogen and fuel cell. The remaining portion of the vehicle fleet, they consist of internal combustion engine (ICE) vehicles, and some HEV/PHEV in the case of light-duty vehicles. As for HGVs the remaining composition are all diesel ICEs.

The assumptions used for the CCC's Balanced Net Zero Pathway for Buildings

The Balanced Net Zero Pathway's four priorities are to deliver on the Government's energy efficiency plans, to scale up the market for heat pumps, to expand the rollout of low carbon district heat networks and to prepare for a potential role for hydrogen in heat production. The BNZP scenario includes a wide range of technologies including low-carbon district heat networks, air and ground source heat pumps, resistive and storage heating, solar thermal, and hydrogen technologies including hydrogen boilers and hydrogen hybrid heat pumps, the removal of biomass burning, as well as switching from gas to electric cooking. The 2019 domestic and commercial combustion sector was disaggregated into residential, non-residential and agricultural combustion sources and was provided by the NAEI (3). By applying specific building decarbonisation policies, described below, we created the residential, commercial and public buildings scenarios in 2030, 2040 and 2050.

Residential building assumptions 2030, 2040 and 2050

Domestic Energy Efficiency measures (such as triple glazing, high levels of airtightness, loft, floor and wall insulation) and **behavior change** (such as turning off lights, pre-heating and smarter heating management) were assumed to deliver an average 12% reduction of heat demand to 18m (64%) homes by 2030, 25m (89%) by 2040 and all residential housing (28.3m) by 2050.

Deployment of these changes follows the CCC's recommendation that social houses and fuel poor housing units receive efficiency measures in priority to homeowner occupied. These measures were applied by country, tenure type (rented social, rented private and owner occupied) using the Domestic Energy Performance Certificate (DEPC) dataset (11,12) and by fuel poverty status (fuel poor and not fuel poor) using the fuel poverty status data (13) and the income deprivation domain score data (14, 15). Note that lighting and appliance efficiency is not included in the figures for reduction in heat demand, since both cause a slight increase, as more efficient appliances produce less heat.

Low Carbon heating: Rapid deployment of heat pumps, electric resistive, solar thermal and hybrid heat pumps with hydrogen or bio boilers were applied to 3.6m (13%) homes in 2030, rising to 14.7m (52%) by 2040 and 22.8m (81%) by 2050. These measures were deployed by country, tenure type, by fuel poverty status (as above) and whether the property is on or off the gas grid, using the DEPC dataset (11, 12). Hybrid heat pumps when operating on electric are assumed to meet 80% of the space heating demand and 80% of hot water demand where a hot water cylinder is installed (0% hot water demand when not installed). The proportion of energy for space heating vs. water heating has been assumed to be 80/20 (16, 2014 and personal communication Simon Rayner at the CCC).

Low carbon District Heat Network (LCDHN) deployment: The district heat networks were deployed using an overview of current heating and cooling demand and supply in the UK combined with a geospatial assessment of the economic potential for district heating as an efficient heating solution (17)

(personal communication, Chris Fairbank at BEIS). LCDHN's were assumed to provide heat to 1.3m (4.5%) homes in 2030, 3.8m (13%) in 2040 and 5.5m (19%) in 2050.

Hydrogen boilers combined with hybrid heat pumps were deployed before gas grid conversion, and were assumed to operate on gas, in hybrid mode, until hydrogen becomes available in 2040 (18). Hydrogen hybrid heat pumps were deployed within LCDHN areas (19, 20; personal communication, Rachel Lee at BEIS). Hydrogen boilers were assumed to eliminate PM emissions and reduce NO_X emission by 66%, based upon typical gas boilers value of 73 mg kWh^{-1} (NAEI) and new hydrogen boiler values of 25 mg kWh^{-1} (21) (personal communication John Foyster at the CCC). It should be also noted that the use of H2 boilers is predicted to be small in Buildings sector. For residential building, the use of H2 boilers in combination with Hybrid Heat Pumps is predicted to be 0.06% in 2030, 1.5% in 2040 and 2.7% in 2050 of the total heat demand for UK homes. For non-residential building sector its 0% in 2030, 2.1% in 2040 and 5% in 2050 of the total heat demand is met by H2 boilers. Although the flame temperature of hydrogen is higher, potentially leading to increased thermal NOx formation, manufacturers have already developed hydrogen boilers with low-NOx emissions through advanced burner technology. For example, work from the UK government's Hy4Heat program has demonstrated the feasibility of these low-NOx hydrogen boilers. These systems are designed with optimized combustion controls that minimize thermal NOx formation, allowing for significant NOx reductions compared to conventional gas boilers.

Bio boilers (bio-oil and bio-LPG systems) were assumed to run at the same efficiency on average (84%) as their fossil fuel counterparts (personal communication, Alex Lochead at BEIS).

New homes were assumed to have heat pumps fitted; to 2.2m new homes by 2030, 3.8m by 2040 rising to 4.7m by 2050 or built within Low carbon District Heat Networks (0.2m by 2030, 0.6m by 2040 and

1.1m by 2050). However, from 2018 to 2024 (inclusive), the CCC BNZP scenario assumed that new homes were built with gas boilers, and that these were replaced with heat pumps 15 years later. As a consequence, gas boilers in 1.6m new homes have been included in the 2030 projections using separate totals for each UK country, and starting from 2033, were assumed to be fully retrofitted by 2039.

Gas cookers were assumed to become electric following the deployment of LCDHNs or Heat pumps because they are unlikely to be economic (personal communication Simon Rayner at the CCC).

Biomass, oil and coal boilers installations were assumed to be phased out by 2028 and gas boiler installations by 2033, assuming a residential boiler lifetime of 15 years (personal communication, Simon Rayner at the CCC). Note hybrid heat pumps with liquid biofuels are permitted in the Balanced Net Zero Pathway scenario.

Commercial and public buildings 2030, 2040 and 2050

Behavioural changes (such as improved energy management and training) and energy efficiency measures (such as improved fabric efficiency, upgrades to lighting and cooling equipment, controls and metering) resulted in a reduction in energy consumption of 26% (across all commercial building) and 24% (across all public building) by 2030 (22; personal communication, Bea Natzler at the CCC). We assumed a reduction in heat demand of 37% (2030), 69.74% (2040) and 100% (2050) based on the CCC BNZP scenario (23), see further details below. This coincided with a rapid deployment of heat pumps: 24% in 2030, 38% in 2040 rising to 52% by 2050.

Low carbon District Heat Networks (LCDHN) deployment resulted in 12% heat demand reduction (across all commercial/public building) in 2030, 29% in 2040 and 42% in 2050. The LCDHN are

assumed to be largely electrified using industrial scale heat pumps drawing on heat sources such as water and waste heat from industry and sewage treatment. LCDHN were distributed around the UK using heat/cooling demand maps of economically viable LCDHN in the same way as for the residential sector. Direct electric deployment using collaboratively optimised distribution of renewal energy resulted in a 0.5% heat demand reduction (across all commercial/public building) in 2040 and 1% by 2050.

Hydrogen boilers were deployed within the same areas as for LCDHN. H_2 boilers reduced heat demand by 2.1% and 5% in 2040 and 2050, respectively. Biomass, oil and coal boiler installations were phased out of by 2025 (public buildings) and 2026 (commercial buildings) and gas boilers by 2030 (public buildings) and 2033 (commercial buildings). We assumed a boiler lifetime of around 20 years (personal communication, Simon Rayner at the CCC). Finally, the BNZP scenario assumes complete phase out of biomass boilers by 2050 with only 1.1% and 0.4% of heat demand still generated from biomass boilers in 2030 in 2040, respectively.

Table S3 UK emissions (in kiloton per year) in 2019, 2030, 2040, and 2050

SNAP sector	2019							2030							2040							2050						
	NH_3	SO_2	NO_X	PM_{10}	$PM_{2.5}$	VOC	CO	NH_3	SO_2	NO_X	PM_{10}	$PM_{2.5}$	VOC	CO	NH_3	SO_2	NO_X	PM_{10}	$PM_{2.5}$	VOC	CO	NH_3	SO_2	NO_X	PM_{10}	$PM_{2.5}$	VOC	CO
1	-	1.4	3.4	0.65	0.64	0.26	2.7	-	0.92	2.8	0.55	0.53	0.19	2.1	-	0.57	1.7	0.42	0.41	0.13	1.3	-	0.026	0.27	0.096	0.093	0.12	0.21
2 BAU	2.6	47.3	55.3	24.4	23.9	50	469	2.9	16.7	55.0	21.5	21.1	47.1	467	2.5	8.6	56.9	18.4	18.0	40.4	467	-	-	-	-	-	-	-
2 BP	-	-	-	-	-	-	-	2.0	16.7	38.2	19.3	18.9	38.8	363	0.81	8.1	17.3	8.4	8.2	16.3	148	0.02	0.37	1.1	0.69	0.69	0.42	4.1
3	0.072	22.1	83.5	14.9	14.4	3.0	70	0.080	21.6	87.4	15.4	14.9	3.2	74.9	0.075	21.3	84.7	11.9	11.5	2.6	72.6	0.14	6.8	52.5	10.2	9.9	4.7	47.4
4	0.02	12.3	4.3	39.2	6.5	39.9	23.1	0.018	13.9	3.5	38.9	6.3	39.5	18.7	0.019	13.3	3.2	39.7	6.2	40.2	16.9	0.02	8.4	2.9	46.4	7.7	39.9	15.1
5	-	0.001	0.16	0.10	0.016	33.4	0.8	-	0	0.083	0.042	0.006	19.6	0.42	-	0	0.042	0.026	0.003	14.1	0.21	-	0	0.003	0.002	0	9.4	0.01
6	1.3	0.026	0.059	2.3	1.5	288	1.8	1.4	0.021	0.05	2.3	1.4	305	1.5	1.5	0.017	0.039	2.4	1.4	327	1.2	1.2	0.017	0.042	2.6	1.5	350	1.2
7 BAU	4.6	1.3	307	19.9	8.8	30.1	230	4.7	1.3	109	18.6	7.2	22.8	66.8	4.7	1.4	74.3	20	7.5	22.6	37.3	-	-	-	-	-	-	-
7 BNZP	-	-	-	-	-	-	-	4.7	1.3	86.3	16.3	6.3	17.8	52.2	4.7	1.4	14.1	15.8	5.7	3.8	6.3	1.6	0.6	1.04	15.9	5.5	0.11	3.3
7 WI	-	-	-	-	-	-	-	4.7	1.3	87.1	16.6	6.4	18.0	52.7	4.7	1.4	20.6	16.4	5.9	5.9	9.6	-	-	-	-	-	-	-
8*	0.041	4.4	101	6.4	6.4	31.6	407	0.041	3.6	82	5.3	5.3	29.1	335	0.043	3.7	83	5.5	5.5	30.1	342	0.048	1.3	29.4	1.9	1.9	14.6	107
8**	0.049	29.3	326	5.3	5.0	9.1	21.7	0.045	14.6	266	4.2	4.0	9.1	17.7	0.044	13.8	229	4.1	3.9	8.8	15.2	0.049	4.7	71	1.4	1.3	4.3	4.8
9	6.6	0.15	0.97	2.5	2.2	6.6	10.9	7.4	0.14	1.1	2.5	2.2	6.2	12.4	7.5	0.14	1.1	2.5	2.3	6.3	12.9	8.7	0.06	0.8	2.1	1.9	6.9	6.0
10	243	-	32.2	16.1	2.9	113	-	244	-	31.6	16.1	2.8	113	-	245	-	31.7	16.1	2.8	113	-	249	-	29.6	16.9	3.0	113	-
Isoprene	-	-	-	-	-	87.1	-	-	-	-	-	-	70.2	-	-	-	-	-	-	90.8	-	-	-	-	-	-	92.1	-

Snap sector description: Snap 1 - Combustion in the production and transformation of energy, Snap 2 - Non-industrial combustion plants, Snap 3 - Industrial combustion plants, Snap 4 - Industrial processes without combustion, Snap 5 - Extraction and distribution of fossil fuels and geothermal energy, Snap 6 - Use of solvents and other products, Snap 7 - Road Transport, Snap 8 - Other mobile sources and machinery, Snap 9 - Waste treatment and disposal, Snap 10 – Agriculture, Snap 11 – Other sources and sinks (nature) is calculated using the CMAQ model.

BAU: Business as usual, BNZP: Balanced Net-Zero pathway, WI: Widespread Innovation

8* Other mobile sources and machinery excluding shipping source

8** Shipping mobile source only

Section S2 Meteorology and air pollution modelling method

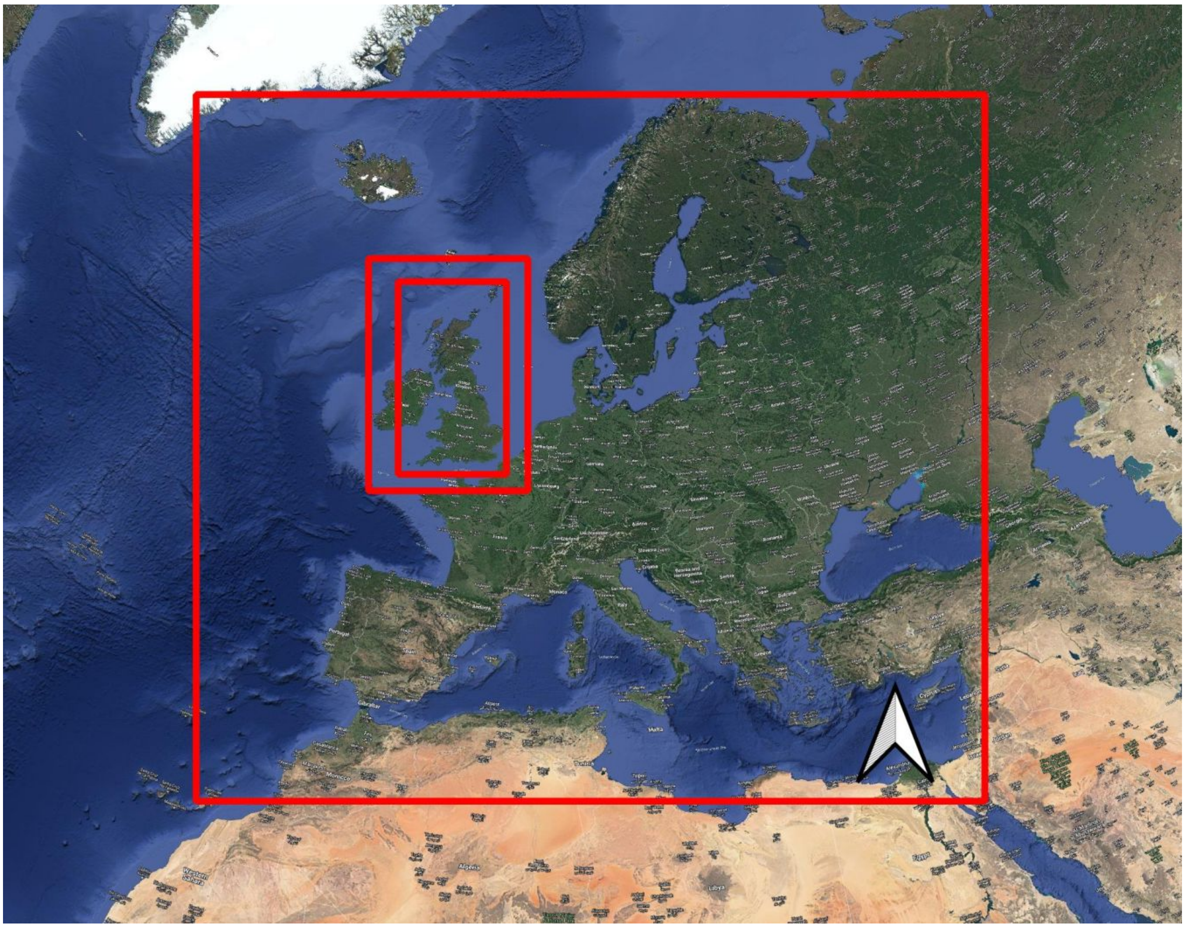

Figure S2 Modelling Domains

Note: The WRF and CMAQ model domains consist of two nested domains covering the UK at horizontal resolutions of 10 km and 2 km, and an outer domain covering Europe at a resolution of 50 km.

Table S4 WRF and CMAQ model configuration

WRF Configuration	
Meteorology model	WRF v4.2
Horizontal resolution	50 x 50km, 10 x 10km, and 2 x 2km
Longwave Radiation	RRTM (24)
Shortwave Radiation	Dudhia scheme (25)
Surface-layer option	MM5 (26)
Land-surface	NOAH (27)
Planetary boundary layer	YSU (28)
Cumulus option	Kain-Fritsch (29)
**CMAQ Configuration**	
Chemistry model	CMAQ v5.4
Horizontal resolution	50 x 50km, 10 x 10km, and 2 x 2km
Vertical Structure	23 vertical layers with 7 layers within 1 km of the ground and the highest being ~15 km above
Gas-phase chemistry	CB6 (30)
Dry deposition	M3dry (31, 32)
Cloud chemistry	KMT2 (33)
Aerosol mechanism	AERO7 (34)
Chemical Solver	Ros3 (35)
Advection Scheme	PPM (36)
Horizontal Diffusion	Multiscale
Vertical Diffusion	ACM2

Section S3 Meteorology and Air quality Models Evaluation

WRF model's performance was evaluated for temperature (T), wind speed (WS), and relative humidity (RH) against observation data at 165 monitoring sites (Figure S3) operated by the UK Met. Office (37). the WRF model shows high accuracy in predicting temperature (T), wind speed (WS), and relative humidity (RH), with correlation coefficients (r) of 0.96 for and 0.80. and 0.79, respectively, accompanied by minimal biases below 10% for all these parameters (Table S5). Although there was a slight underestimation in both WS and T, as indicated by negative mean biases. Overall, the high correlation values across all parameters underscore the model's effectiveness in capturing the overall meteorological conditions. Scatter plots of annual average modelled vs observed values for temperature, wind speed, and relative humidity are also shown in Figures S5 to S7.

The CMAQ-urban model's performance for NO_2, PM_{10}, $PM_{2.5}$, and O_3 was evaluated against observational data from a wide network of monitoring sites (Figure S4) from the Automatic Urban and Rural Network (AURN), the Scottish air quality network (SAQN) the Wales air quality network (WAQN), and the Northern Ireland air quality network (NI), all maintained by Department for Environment, Food, and Rural Affairs (Defra) (38). In addition, data from the London air quality network (LAQN) run by Imperial College London (39), and the Air Quality England database (AQE) collected by Ricardo Energy & Environment (40) were used. The summary of the model's performance statistics is summarised in Table S6. For NO_2, a slight underestimation is noted with a mean bias (MB) of -1.7 mg m^{-3}, while the mean gross error (MGE) of 6.2 mg m^{-3} signifies a moderate scatter (Figure S8) which is more pronounced at roadside locations than at urban background sites. The normalized metrics, normalised mean bias (NMB) and normalised mean gross error (NMGE), are indicative of a well-calibrated model given their values of -0.1 and 0.21, respectively. The model's precision is further substantiated by an RMSE of 9.7 mg m^{-3} and a robust correlation coefficient (r) of 0.79, affirming the model's competence in simulating NO_2 concentations. In the case of PM_{10}, the model tends to overpredict slightly with a MB of 1.7, though this is balanced by a MGE of 3.1, suggesting good model precision. The NMB and NMGE were relatively low at 0.1 and 0.2, respectively. With an RMSE of 3.9 and a moderate correlation coefficient (r) of 0.66, the statistics underscore an overall acceptable performance for PM_{10} modelling. Like NO_2, the modelled PM_{10} is more scattered at roadside location compared to urban background sites (Figure S9). The performance of the model for $PM_{2.5}$ suggests that the model underestimates the mean $PM_{2.5}$ concentration slightly with a MB of -0.3 mg m^{-3}. This is also confirmed by an MGE of 1.3 mg m^{-3} and NMB of -0.03. The NMGE of 0.13 and an RMSE of 1.7 mg m^{-3} reflect an acceptable performance in the $PM_{2.5}$ predictions. A strong correlation coefficient (r) of 0.71 further corroborates the model's accuracy for $PM_{2.5}$ (Figure S10). Similarly, the model evaluation for O_3 revealed an acceptable degree of accuracy with an MB of -4 mg m^{-3}, suggesting the model slightly underpredicts O_3 concentrations. The MGE of 5 mg m^{-3} and a relatively low NMB of -0.07 imply that the model's O_3 predictions, while variable, remain within a reasonable error margin.

A strong correlation coefficient (r) of 0.88 suggest a competent performance in O_3 predictions. This is confirmed by a relatively low scattered in modelled O_3 as can be seen in Figure S11.

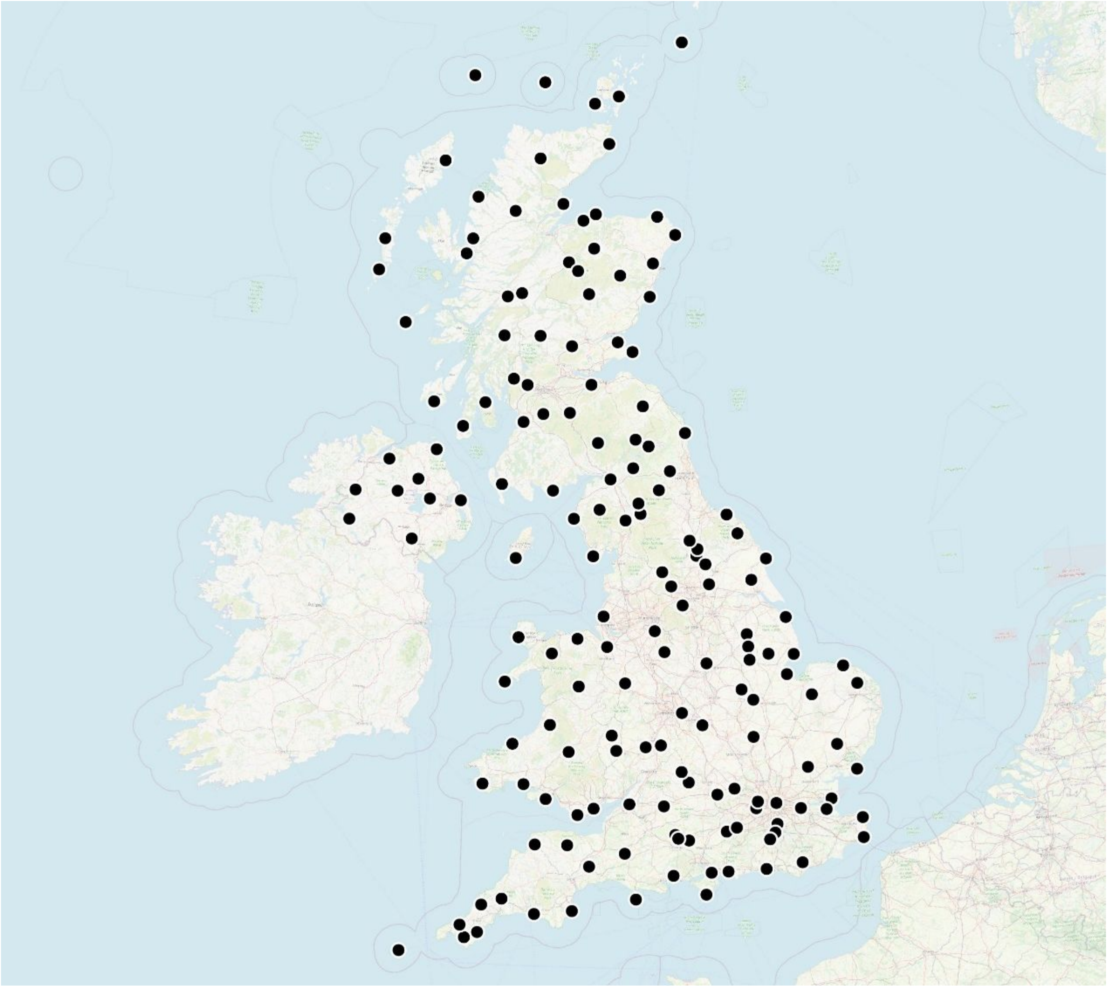

Figure S3 Location of meteorology monitoring sties used for WRF model performance evaluation.

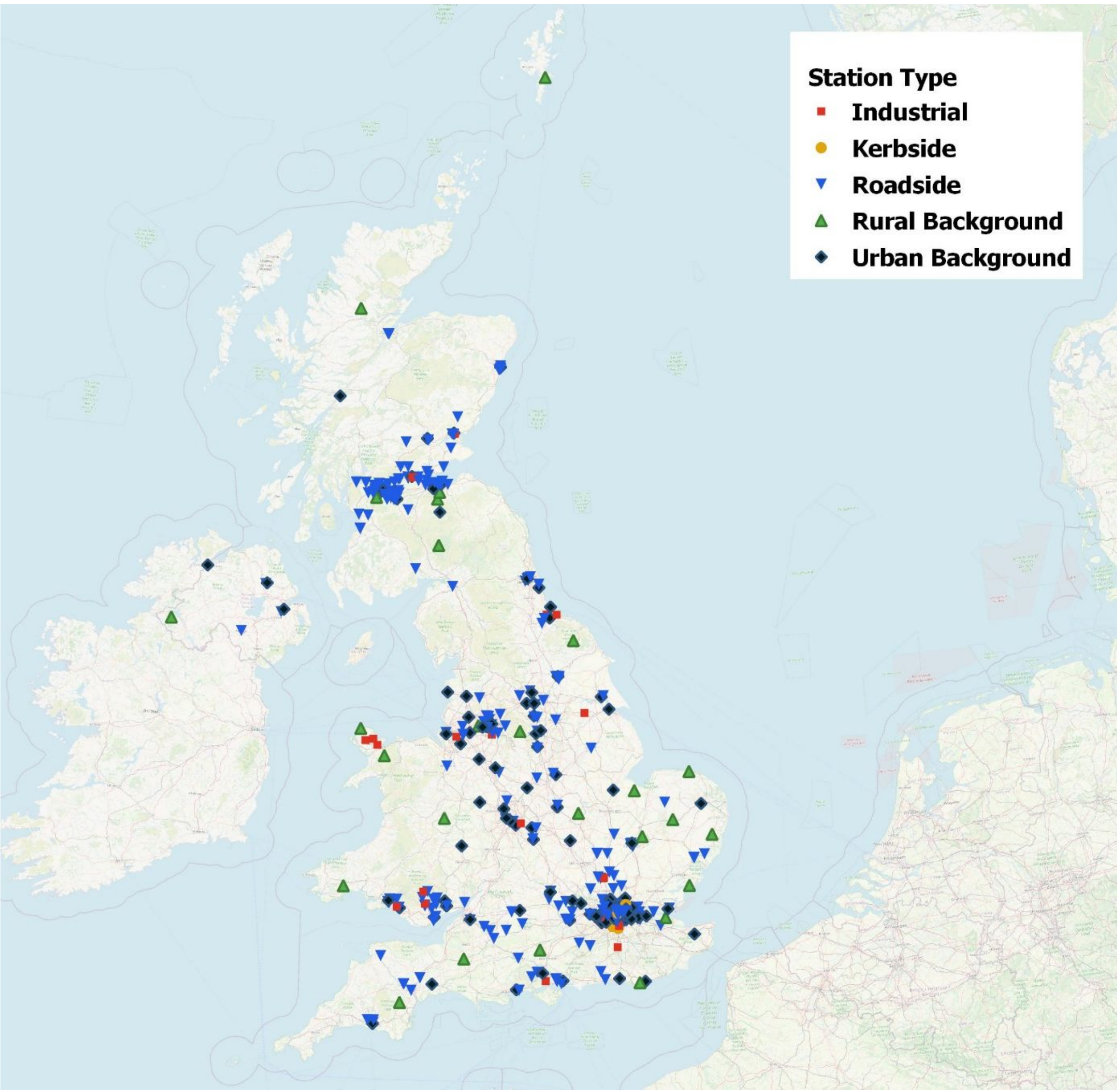

Figure S4 Location and type of air quality network monitoring sties used for CMAQ-urban model performance evaluation.

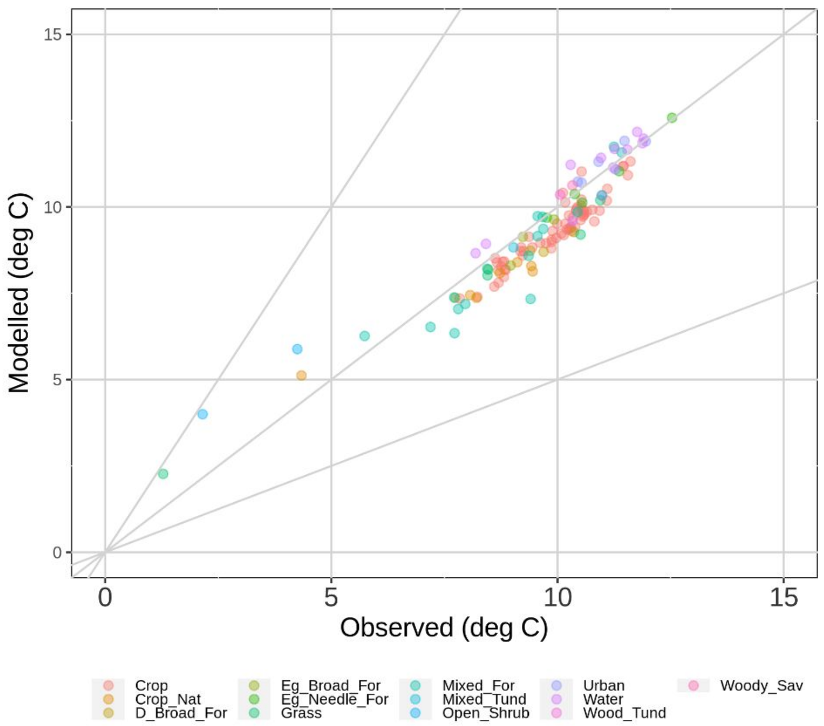

Figure S5 Modelled vs observed annual average of temperature across all UK sites for 2019. Colors of each marker denotes the Land use type associated with each monitoring site.

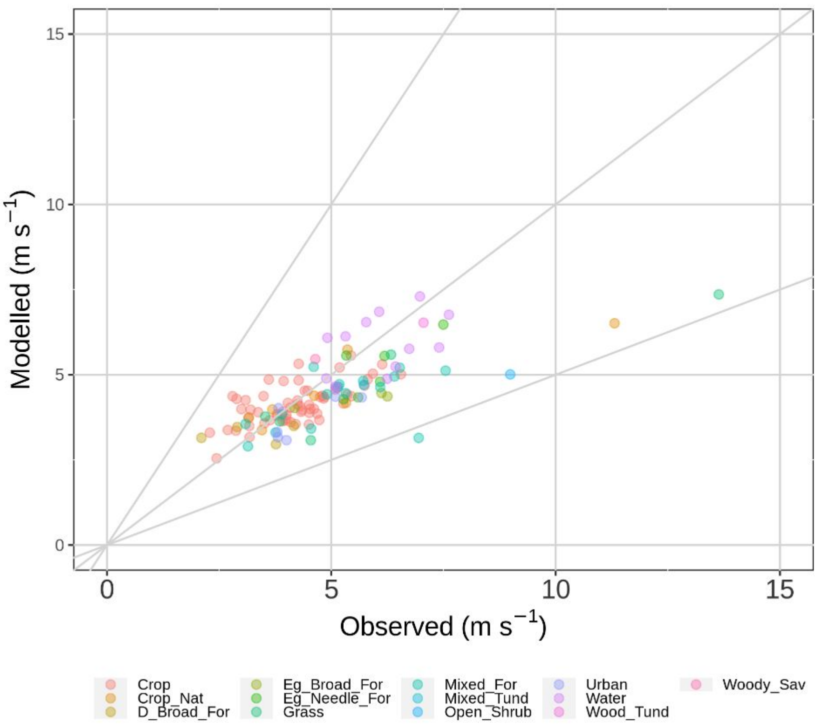

Figure S6 Modelled vs observed annual average of wind speed across all UK sites for 2019.

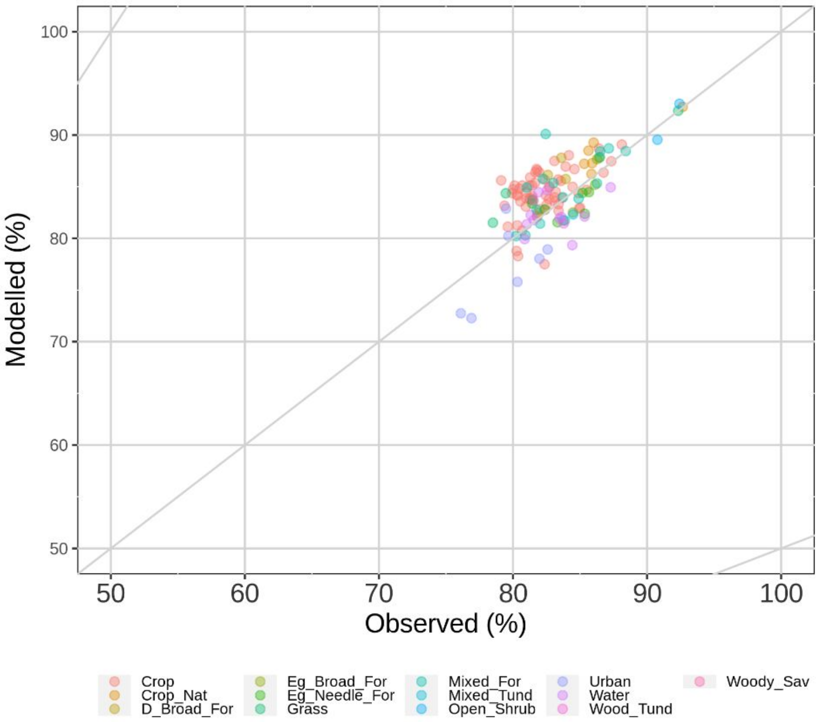

Figure S7 Modelled vs observed annual average of relative humidity across all UK sites for 2019.

Table S5 WRF Model Performance Statistics

Parameter	FAC2	Mean (Obs.)	Mean (Mod.)	MB	MGE	NMB	NMGE	RMSE	r	COE
RH (%)	1.0	82.7	83.9	1.10	6.50	1.40	7.90	8.70	0.79	0.39
T (˚C)	0.91	9.8	9.4	-0.40	1.30	-4.30	13.10	1.70	0.96	0.71
WS (m s^{-1})	0.86	4.8	4.4	-0.40	1.40	-8.80	29.90	2.0	0.80	0.42

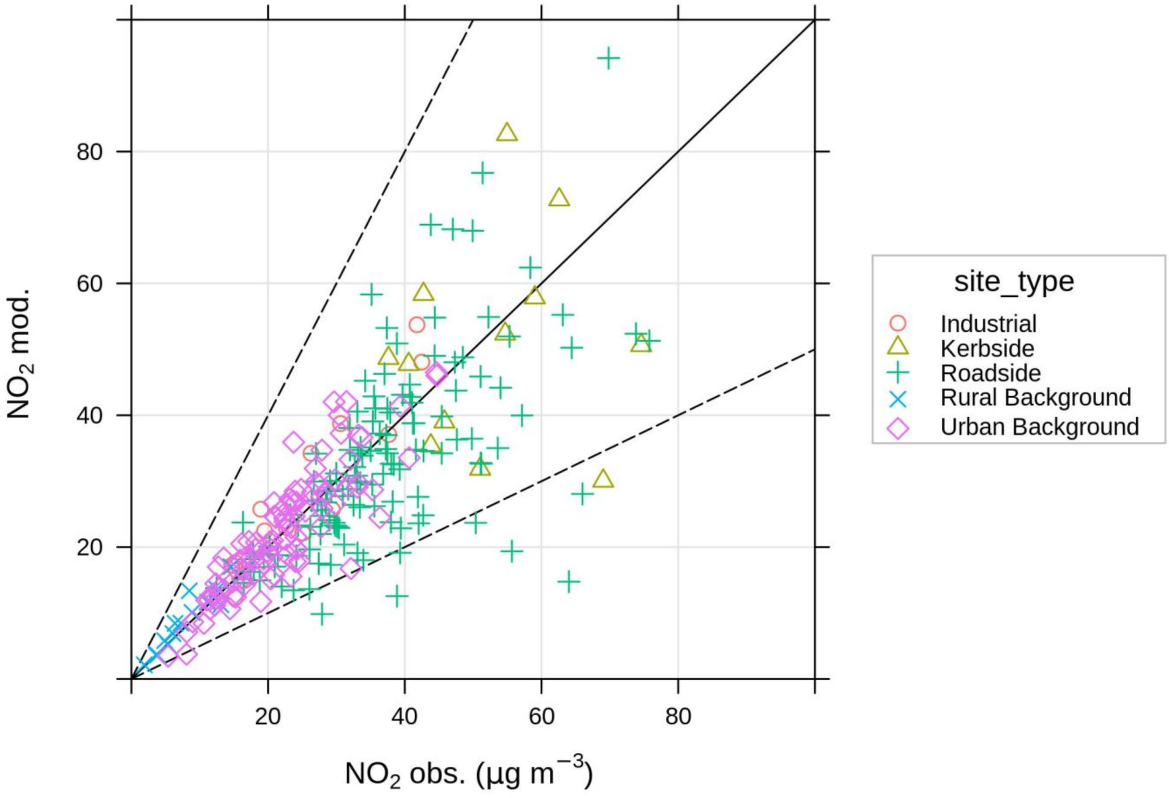

Figure S8 Modelled vs observed annual average of NO_2 concentrations across all UK sites for 2019.

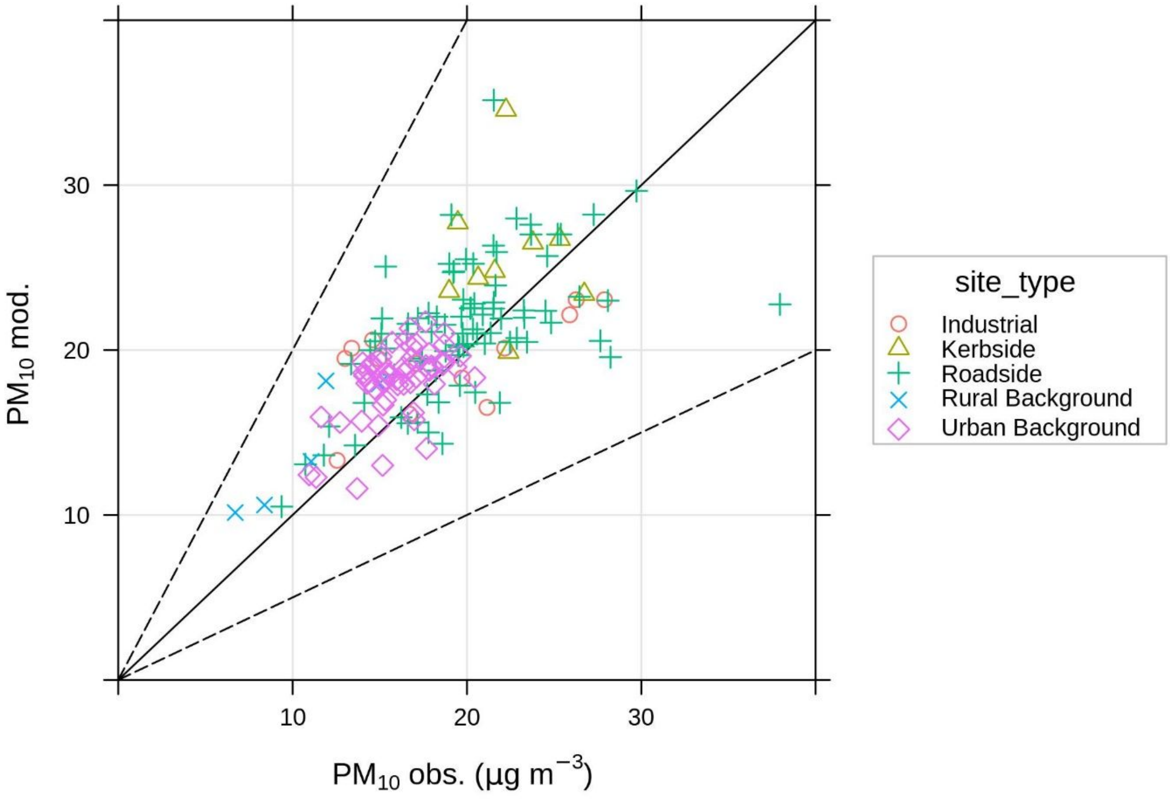

Figure S9 Modelled vs observed annual average of PM_{10} concentrations across all UK sites for 2019.

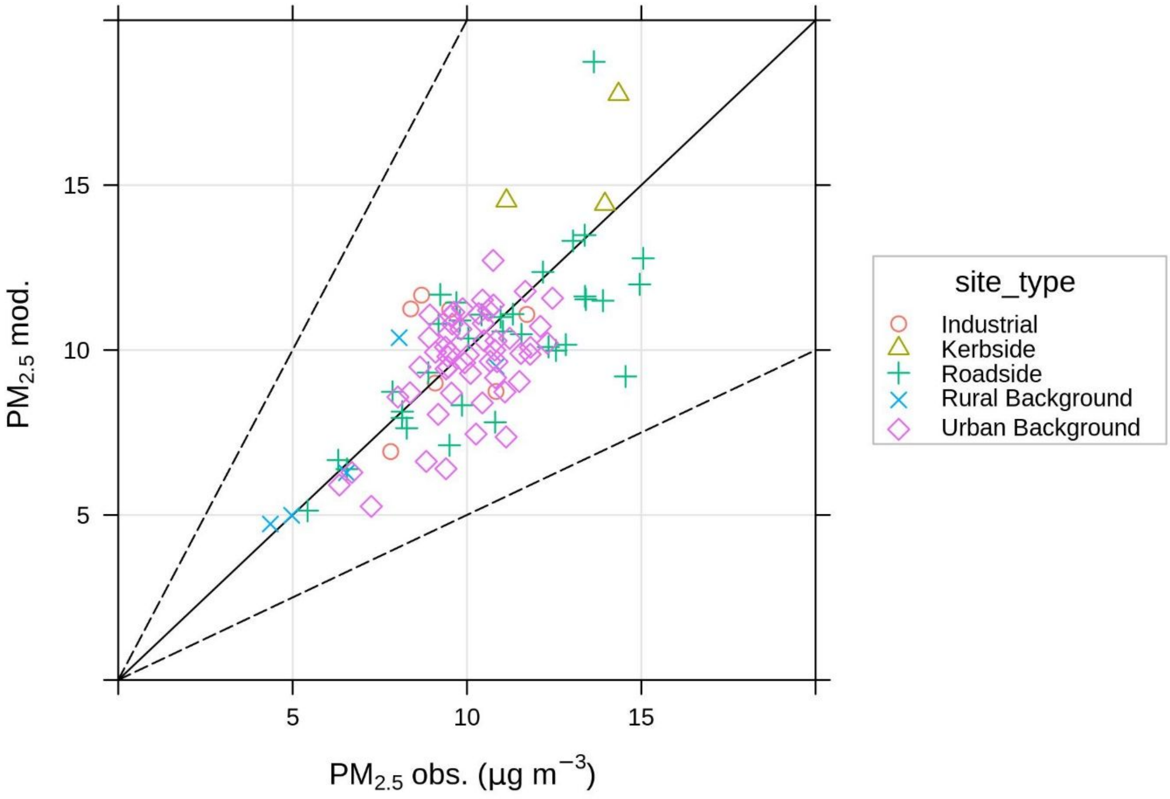

Figure S10 Modelled vs observed annual average of PM_{25} concentrations across all UK sites for 2019.

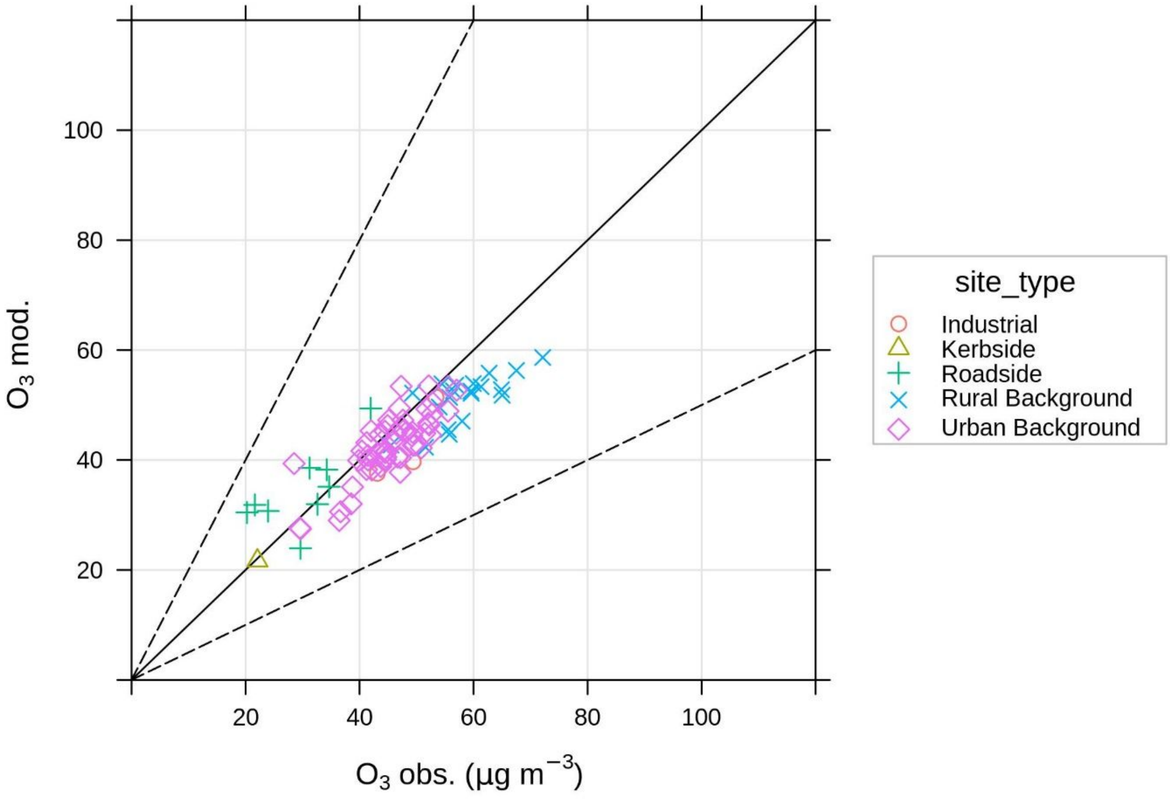

Figure S11 Model vs observed annual average of 8-hour daily maximum O_3 concentrations across all UK sites for 2019.

Table S6 CMAQ-URBAN model performance statistics

Site Type	n	FAC2	MB (mg m^{-3})	MGE (mg m^{-3})	NMB	NMGE	RMSE (mg m^{-3})	r	COE	IOA
					**NO_2**					
UB	95	0.99	0.16	3.26	0.01	0.15	4.44	0.88	0.49	0.75
RD	126	0.94	-3.89	8.45	-0.11	0.23	12.09	0.65	0.12	0.56
RB	15	1.00	0.87	1.17	0.12	0.16	1.70	0.94	0.63	0.82
IND	15	1.00	2.55	4.09	0.10	0.17	5.20	0.94	0.49	0.74
KB	12	0.92	-2.43	14.37	-0.05	0.27	17.96	0.12	-0.52	0.24
					**PM_{10}**					
UB	56	1.00	2.05	2.50	0.13	0.16	2.87	0.55	-0.53	0.24
RD	84	1.00	1.39	3.22	0.07	0.16	4.26	0.56	0.01	0.50
RB	5	1.00	3.41	3.41	0.32	0.32	3.72	0.91	-0.39	0.30
IND	13	1.00	0.47	3.62	0.02	0.19	4.16	0.61	0.21	0.61
KB	9	1.00	3.37	4.67	0.15	0.21	5.69	-0.03	-1.38	-0.16
					**$PM_{2.5}$**					
UB	52	1.00	-0.39	1.18	-0.04	0.12	1.44	0.55	-0.16	0.42
RD	34	1.00	-0.56	1.45	-0.05	0.13	1.98	0.71	0.31	0.66
RB	5	1.00	0.22	0.86	0.03	0.12	1.21	0.87	0.57	0.78
IND	7	1.00	0.55	1.61	0.06	0.17	1.91	0.24	-0.50	0.25
KB	3	1.00	2.42	2.42	0.18	0.18	2.79	0.57	-0.81	0.10
					**O_3**					
UB	54	1.00	-2.69	3.92	-0.06	0.09	4.64	0.81	0.17	0.59
RD	9	1.00	4.44	5.85	0.15	0.20	6.76	0.71	-0.07	0.46
RB	20	1.00	-7.15	7.44	-0.12	0.13	8.34	0.71	-0.55	0.22
IND	3	1.00	-5.74	5.74	-0.12	0.12	6.55	0.87	-0.56	0.22

Note: UB: Urban Background; **RD:** Roadside; **RB:** Rural Background; **IND:** Industrial **KB:** Kerbside; **MB**: Mean bias; **MGE**: Mean gross error; **NMB**: Normalised mean bias; **NMGE**: normalised mean gross error; **RMSE**; Root mean square error; **r**: Correlation coefficient; **COE**: Coefficient of efficiency; **IOA**: Index of agreement

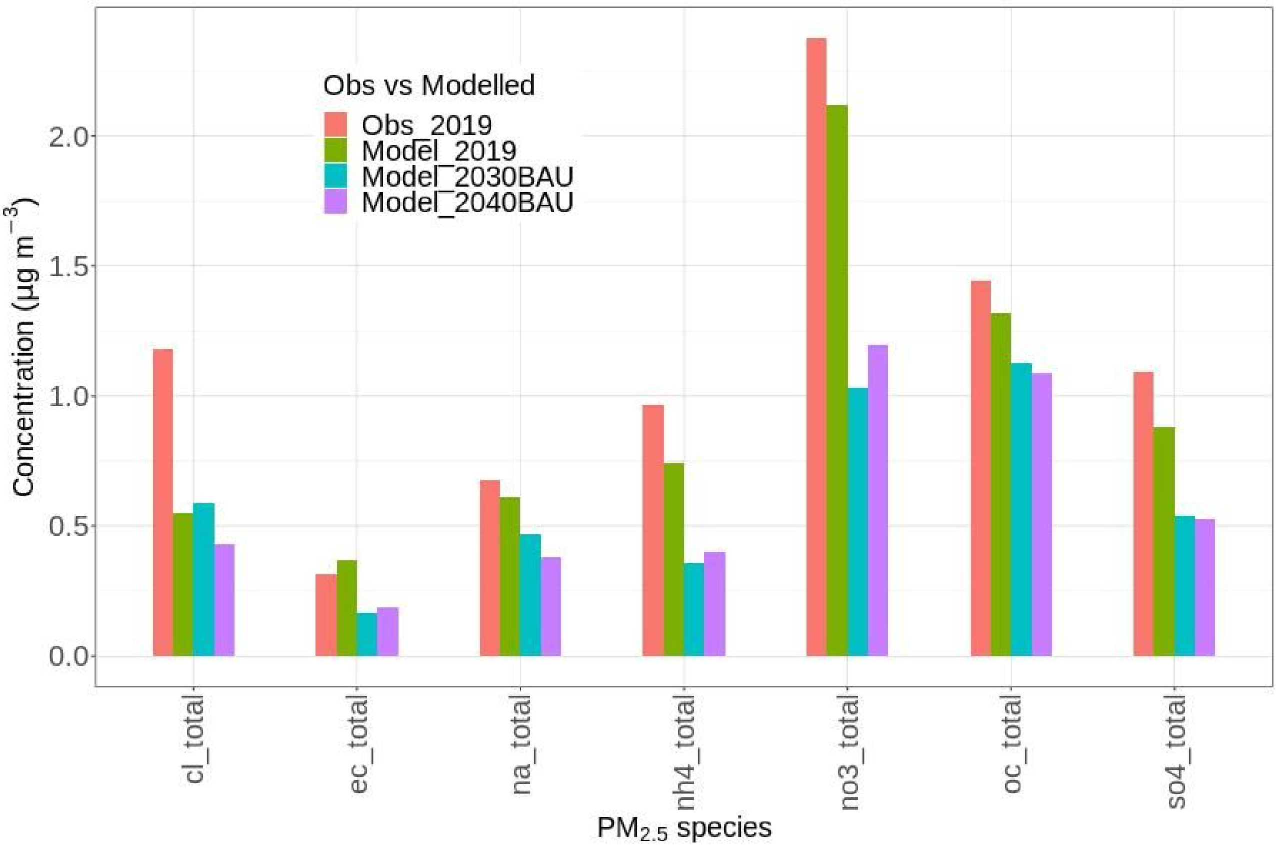

Figure S12 Changes in annual average concentration of $PM_{2.5}$ components between observed in 2019, and modelled for 2019, 2030BAU, and 2040BAU for Chloride (CL), Elemental Carbon (EC), Sodium (NA), Ammonia (NH_4+), Nitrate (NO_3^-), Organic Carbon (OC), and Sulphate (SO_4^{2-})

Section S4 Supplementary modelling results

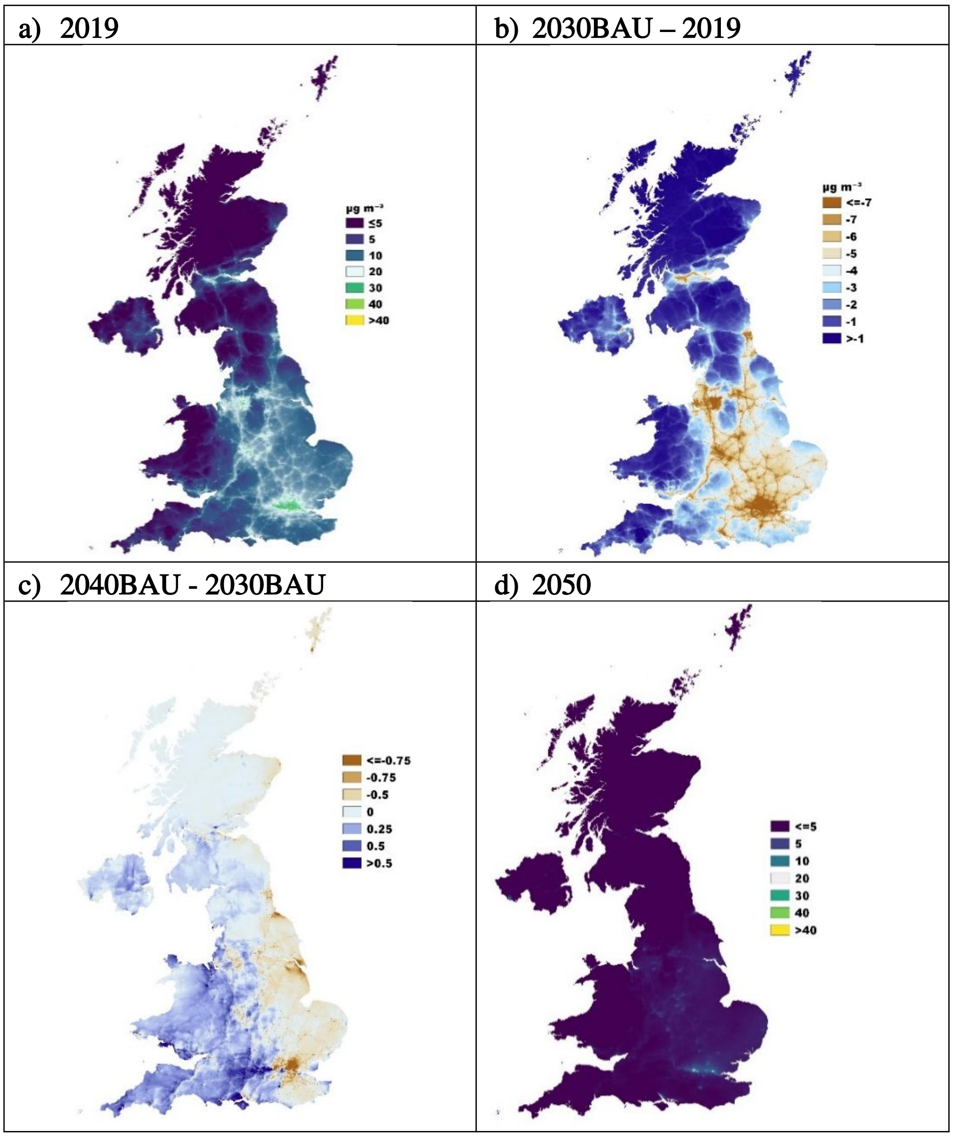

Figure S13 CMAQ-urban UK model results for annual average NO_2 concentrations in 2019 (a), difference between 2030BAU and 2019 (b), difference between 2040BAU and 2030BAU (c), and annual average in 2050 (d)

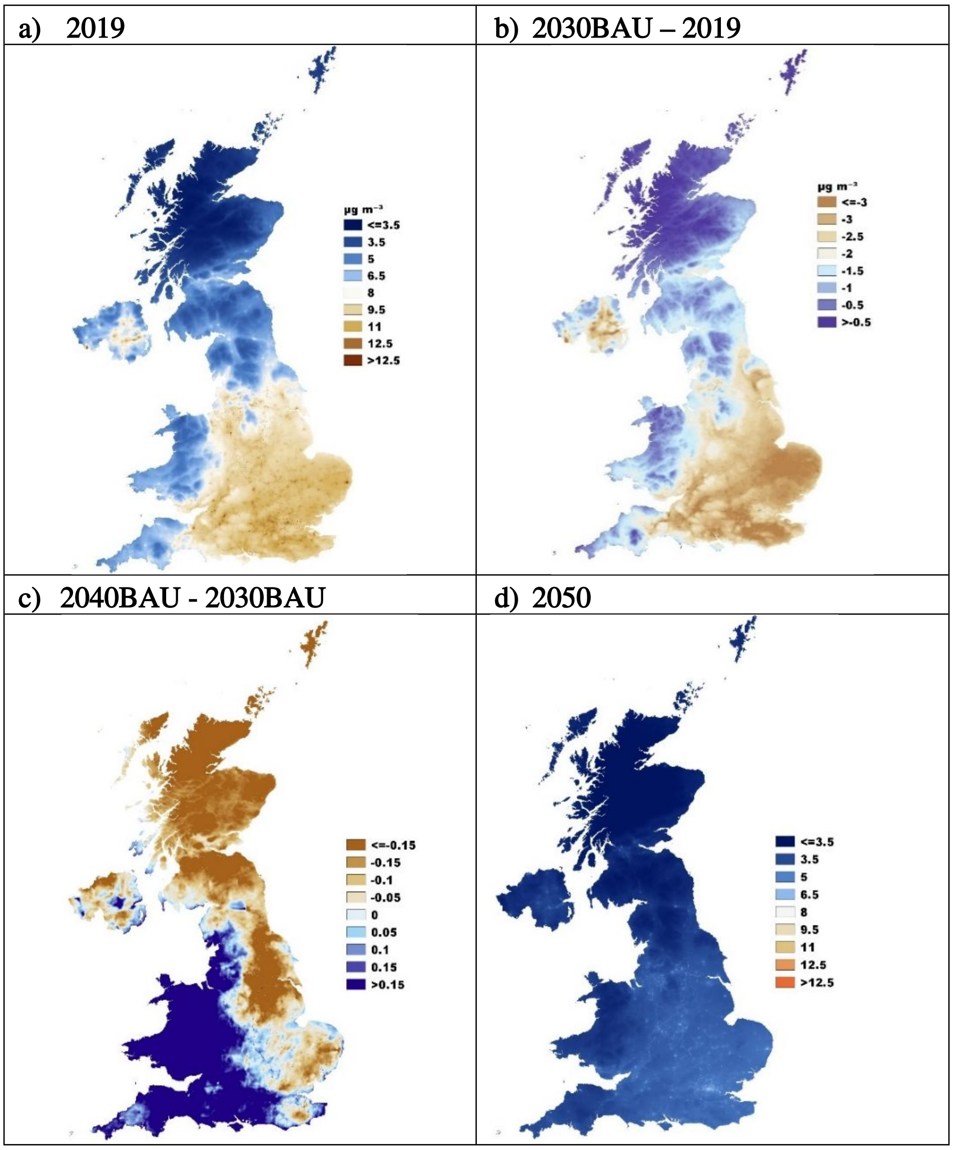

Figure S14 CMAQ-urban UK model results for annual average $PM_{2.5}$ concentrations in 2019 (a), difference between 2030BAU and 2019 (b), difference between 2040BAU and 2030BAU (c), and annual average in 2050 (d)

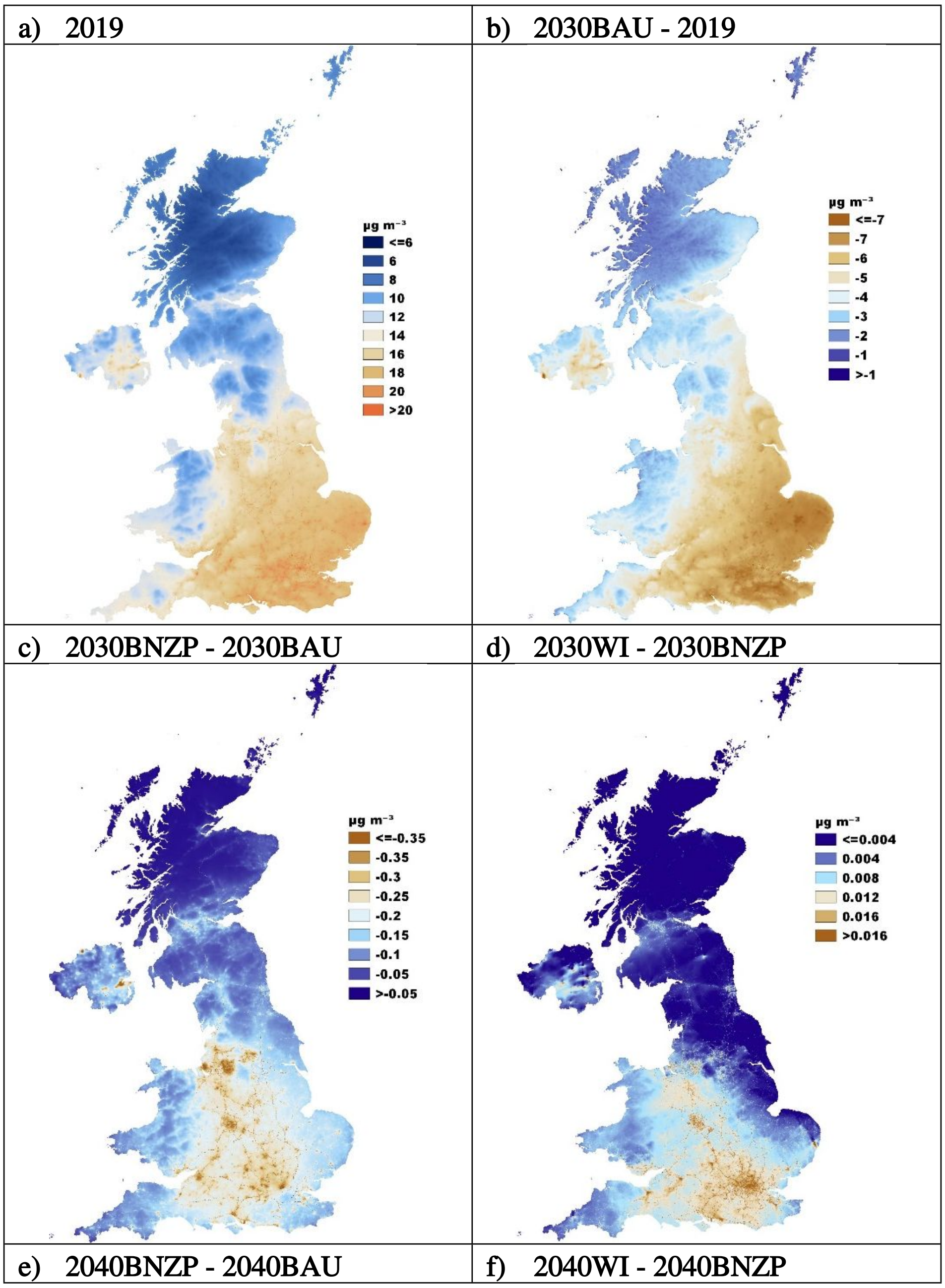
a) 2019
b) 2030BAU - 2019
µg m⁻³
<=6
6
8
10
12
14
16
18
20
>20
µg m⁻³
<=-7
-7
-6
-5
-4
-3
-2
-1
>-1
c) 2030BNZP - 2030BAU
d) 2030WI - 2030BNZP
µg m⁻³
<=-0.35
-0.35
-0.3
-0.25
-0.2
-0.15
-0.1
-0.05
>-0.05
µg m⁻³
<=0.004
0.004
0.008
0.012
0.016
>0.016
e) 2040BNZP - 2040BAU
f) 2040WI - 2040BNZP

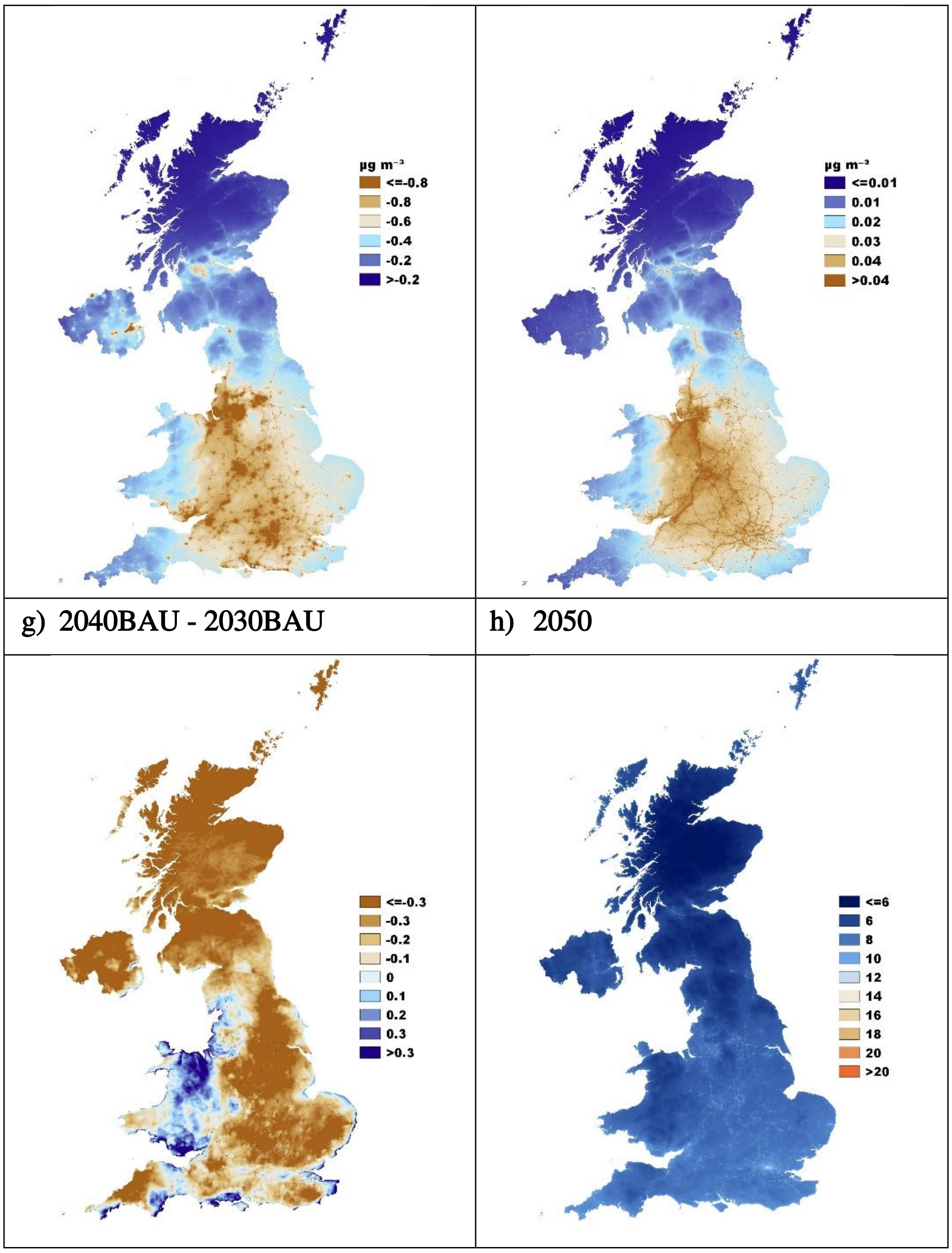

Figure S15 CMAQ-urban UK model results for annual average PM_{10} concentrations in 2019 (a), difference between 2030BAU and 2019 (b), difference between 2030BNZP and 2030BAU (c), difference between 2030WI and 2030BNZP (d), difference between 2040BNZP and 2040BAU (e), difference between 2040WI and 2040BNZP (f), difference between 2040BAU and 2030BAU (g), and annual average concentrations in 2050 (h)

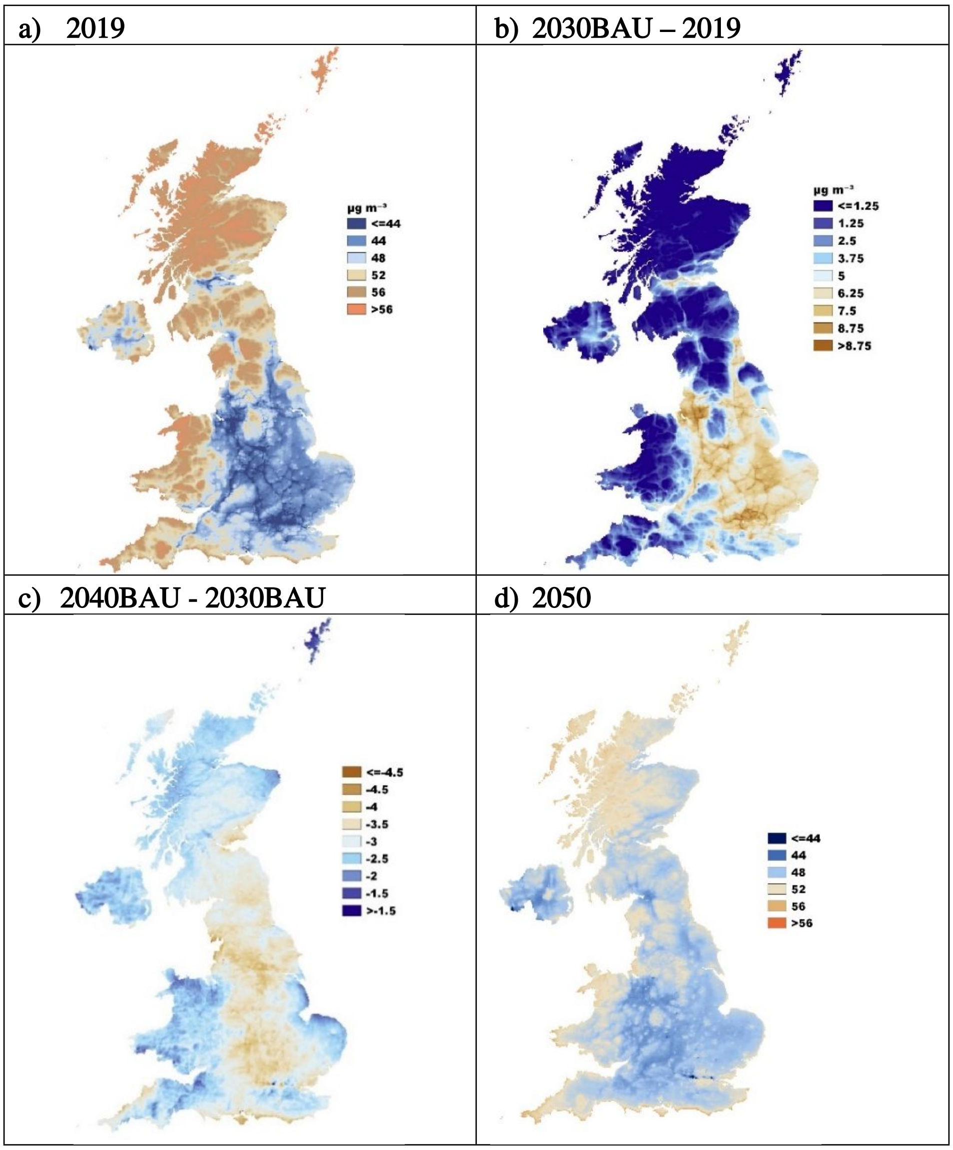
a) 2019
b) 2030BAU – 2019
c) 2040BAU - 2030BAU
d) 2050
µg m⁻³
<=44
44
48
52
56
>56
µg m⁻³
<=1.25
1.25
2.5
3.75
5
6.25
7.5
8.75
>8.75
<=-4.5
-4.5
-4
-3.5
-3
-2.5
-2
-1.5
>-1.5
<=44
44
48
52
56
>56

Figure S16 CMAQ-urban UK model results for annual average O_3 concentrations in 2019 (a), difference between 2030BAU and 2019 (b), difference between 2040BAU and 2030BAU (c), and annual average in 2050 (d)

Table S7 Population weighted average concentration by country

Country	2019	2030BAU	2030BNZBP	2030WI	2040BAU	2040BZNP	2040WI	2050
				NO_2 (µg m^{-3})				
England	17.05	10.65	9.81	9.85	10.47	7.95	8.11	3.50
Wales	8.90	5.79	5.24	5.26	6.06	4.61	4.67	2.03
Scotland	9.49	6.02	5.31	5.33	5.93	4.18	4.25	1.69
N. Ireland	7.68	4.77	4.62	4.63	4.83	4.11	4.15	1.87
UK	15.78	9.88	9.08	9.12	9.73	7.38	7.52	3.24
				$PM_{2.5}$ (µg m^{-3})				
England	9.60	7.16	6.96	6.97	7.25	6.56	6.58	4.40
Wales	7.61	5.98	5.83	5.84	6.60	5.96	5.99	3.81
Scotland	5.47	4.17	4.08	4.08	4.0	3.66	3.67	2.77
N. Ireland	8.19	5.64	5.45	5.45	5.65	5.07	5.08	3.35
UK	9.13	6.82	6.63	6.64	6.91	6.25	6.28	4.21
				PM_{10} (µg m^{-3})				
England	17.21	11.30	11.04	11.05	11.14	10.29	10.33	8.35
Wales	14.53	10.32	10.12	10.13	10.66	9.86	9.90	8.12

Scotland	11.25	7.51	7.37	7.38	7.10	6.65	6.67	6.15
N. Ireland	14.49	9.53	9.28	9.29	9.21	8.52	8.53	7.09
UK	16.52	10.89	10.11	10.13	10.73	9.86	9.96	8.12
**8-hour max. O_3 ($\mu g\ m^{-3}$)**								
England	43.01	45.56	45.79	45.78	43.42	43.89	43.88	41.37
Wales	48.39	49.96	50.01	50.02	48.55	48.44	48.46	44.74
Scotland	56.79	58.90	59.17	59.17	55.88	56.44	56.45	53.93
N. Ireland	42.64	43.89	43.84	43.84	41.70	41.64	41.65	39.70
UK	44.39	46.82	47.03	47.03	44.63	45.07	45.06	42.51

a) Daily Maximum Temperature

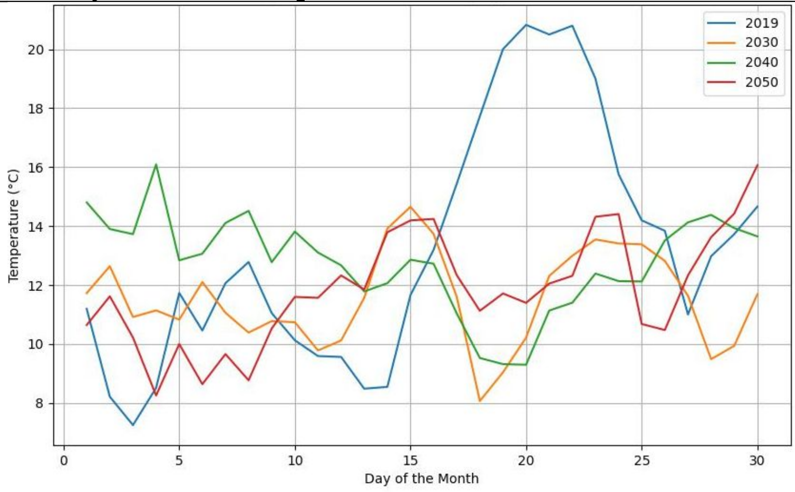

b) Daily Maximum Ozone

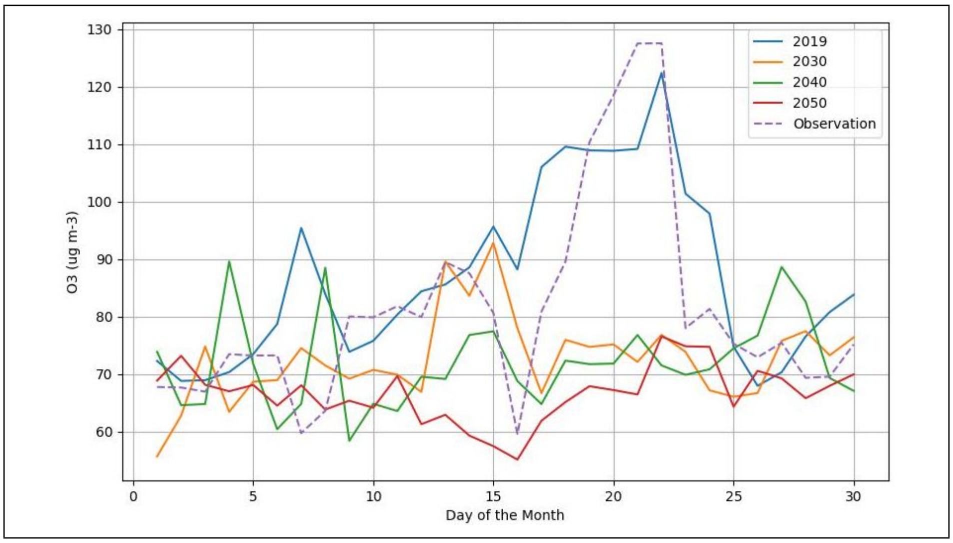

Figure S17 Time series of daily maximum temperature (a), and daily maximum O_3 (b) for April, averaged at monitoring sites. Observed for 2019 (dashed) vs modelled for 2019 (blue), modelled for 2030BAU (yellow), modelled for 2040BAU (green), and modelled for 2050 (red).

c) Daily Maximum Temperature

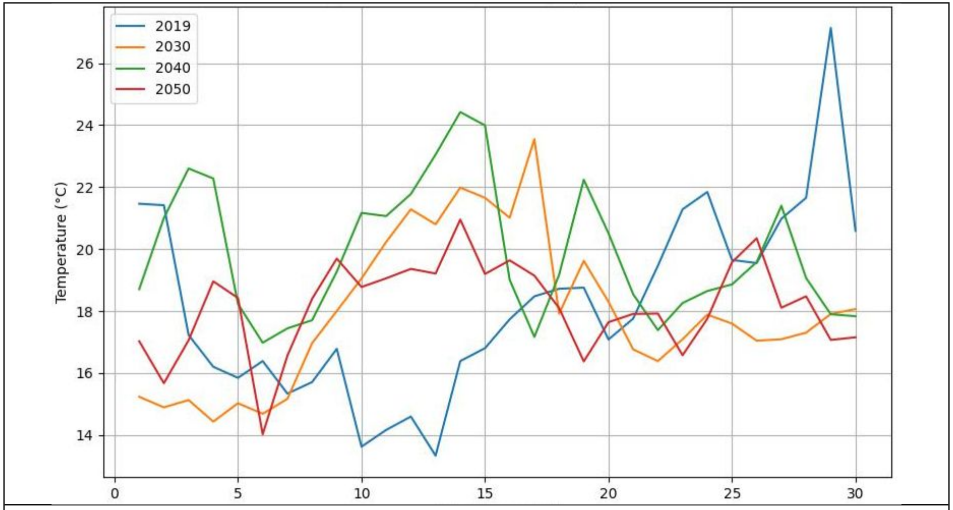

d) Daily Maximum Ozone

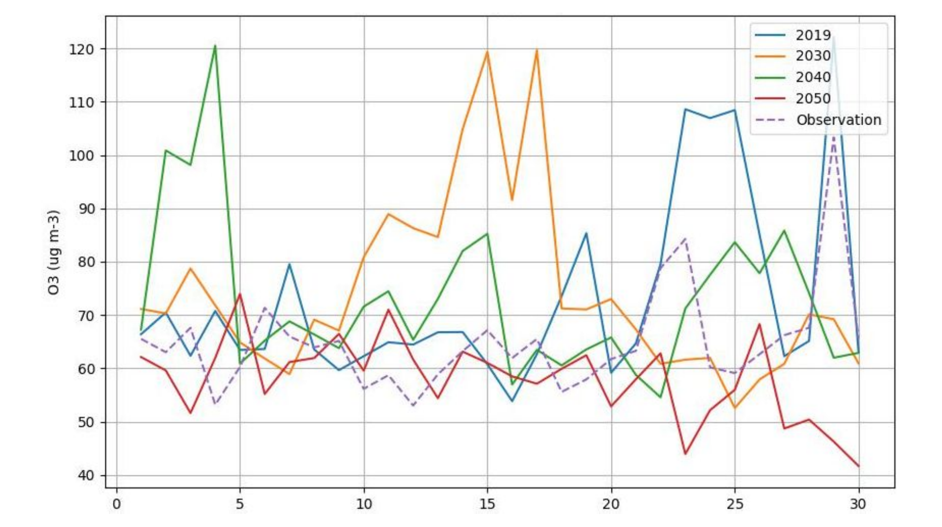

Figure S18 Time series of daily maximum temperature (a), and daily maximum O_3 (b) for June, averaged at monitoring sites. Observed for 2019 (dashed) vs modelled for 2019 (blue), modelled for 2030BAU (yellow), modelled for 2040BAU (green), and modelled for 2050 (red).

a) Daily Maximum Temperature

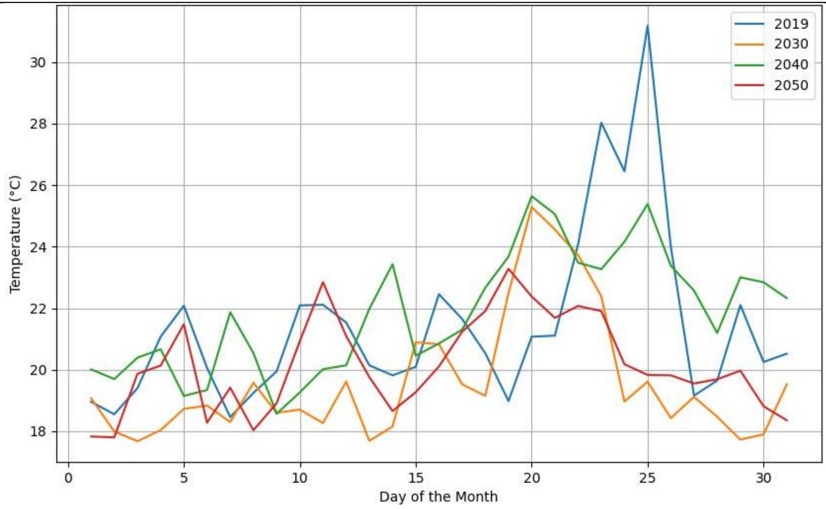

b) Daily Maximum Ozone

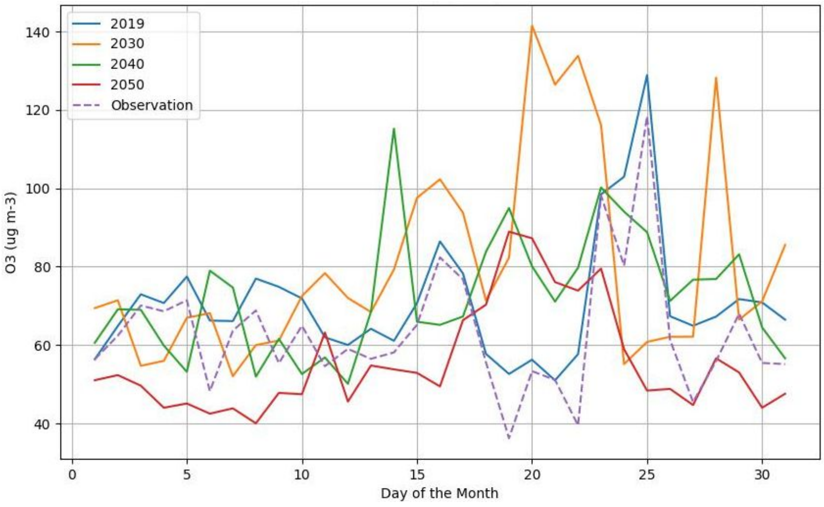

Figure S19 Time series of daily maximum temperature (a), and daily maximum O_3 (b) for July, averaged at monitoring sites. Observed for 2019 (dashed) vs modelled for 2019 (blue), modelled for 2030BAU (yellow), modelled for 2040BAU (green), and modelled for 2050 (red).

a) Daily Maximum Temperature

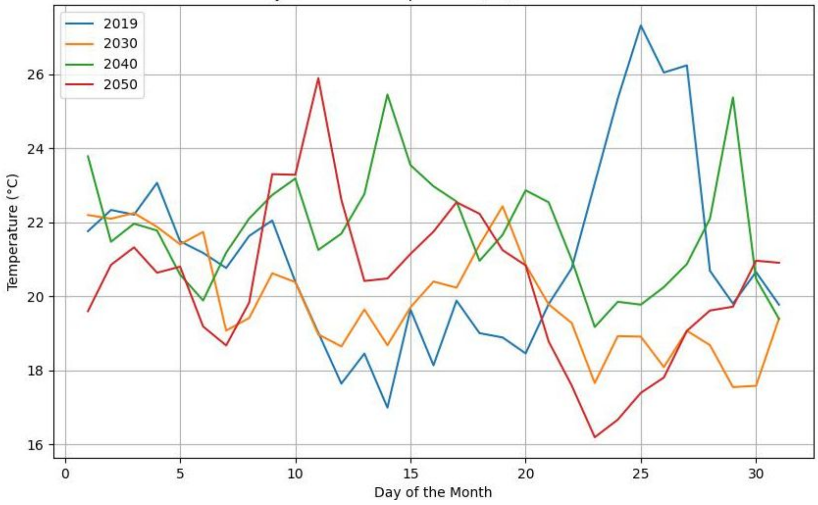

b) Daily Maximum Ozone

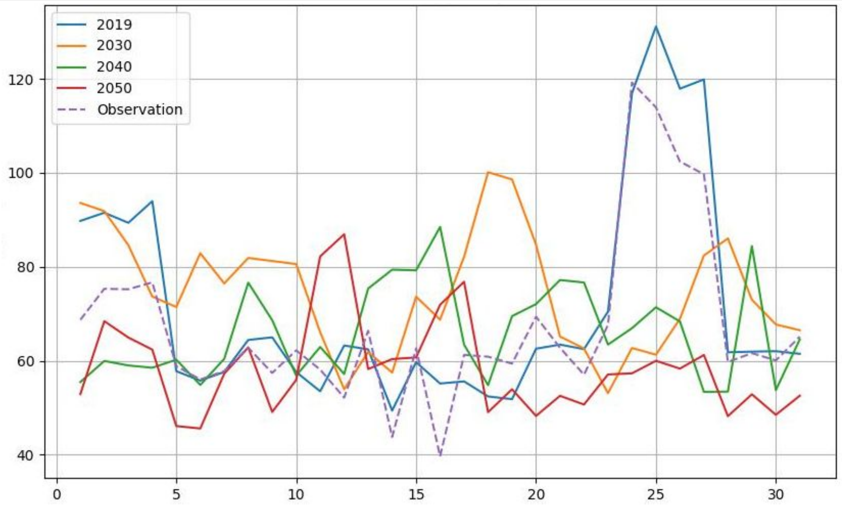

Figure S20 Time series of daily maximum temperature (a), and daily maximum O_3 (b) for August, averaged at monitoring sites. Observed for 2019 (dashed) vs modelled for 2019 (blue), modelled for 2030BAU (yellow), modelled for 2040BAU (green), and modelled for 2050 (red).

Population Weighted Average Concentration (PWAC)

Population in 2019 and death data (averaged across three years to give a more stable representation of typical levels of mortality around 2019) were used to produce a 2019 lifetable by gender and single year of age at wards and local authorities level as the starting point for the life-table analysis (used for the health impact assessment). The 2019 population data at wards level was used to represent the population at local authority level in 2019 and for the PWAC calculations to represent the average Wards concentration at local authority level. We used 2019 population data to calculate PWAC for future years. However, we also conducted a sensitivity analysis using 2019 and projected future population data. Populations for future years were obtained from the life table runs (necessary for the health impact calculations). Life table calculations were programmed based on the methods used in the standard IOMLIFET spreadsheets and included deaths data, birth Projections, mortality improvements, extension to 2154, adjustment of the baseline hazard rates over time according to projected mortality rate improvements and inclusion of changes in numbers of births over time The results indicated less than 2% change in PWAC values for all pollutants between the two approaches, suggesting that population projections do not significantly impact the concentration trends or conclusions drawn in our study. Further details (see population data description below) and full method will be disseminated in subsequent publications of the health (and economic) impact assessment of air pollution.

2019 Population data at ward level

For England and Wales, the population data for the year 2019 has been obtained from ONS by gender and by single year of age at Ward level.

For Scotland, the population data for the year 2019 has been obtained from National Records of Scotland by gender and by single year of age at Ward level using the Electoral Wards Version2019.

For Northern Ireland, the population data for the year 2019 has been obtained from NISRA by gender and by broad age bands (0-15, 16-39, 40-64 and 65+) at Ward level. The population was further redistributed by gender and by single year of age at Ward level using data for the year 2019 obtained from NISRA by gender and by single year of age at Administrative Areas.

a) NO_2 (2040BNZP – 2040BAU)

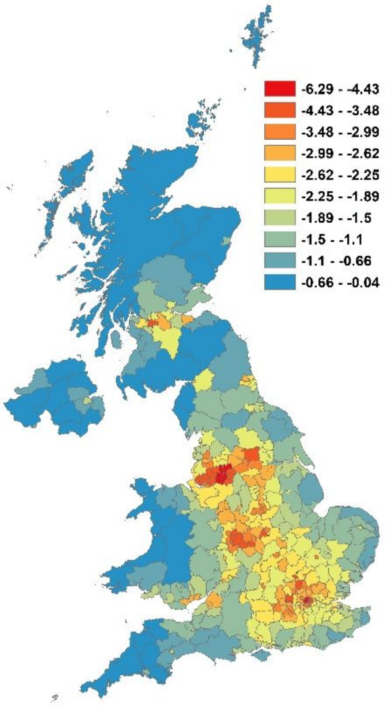

b) $PM_{2.5}$ (2040BNZP – 2040BAU)

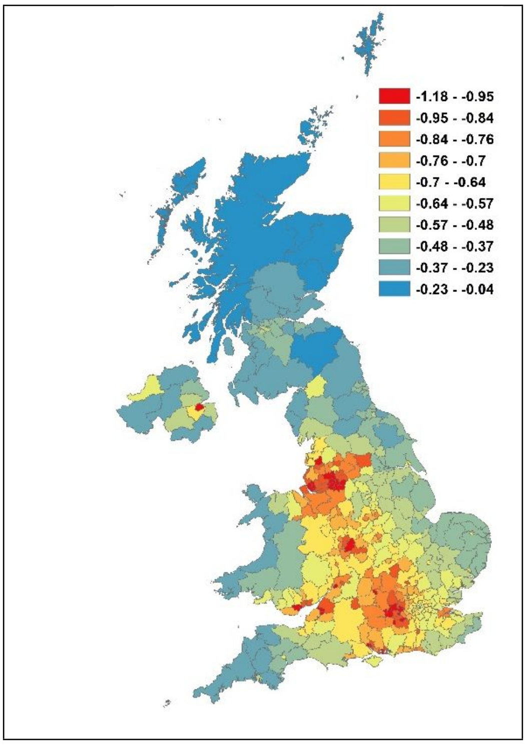

Figure S21 NO_2 and $PM_{2.5}$ PWAC difference maps for each of the UK's local authorities.

a) NO_2

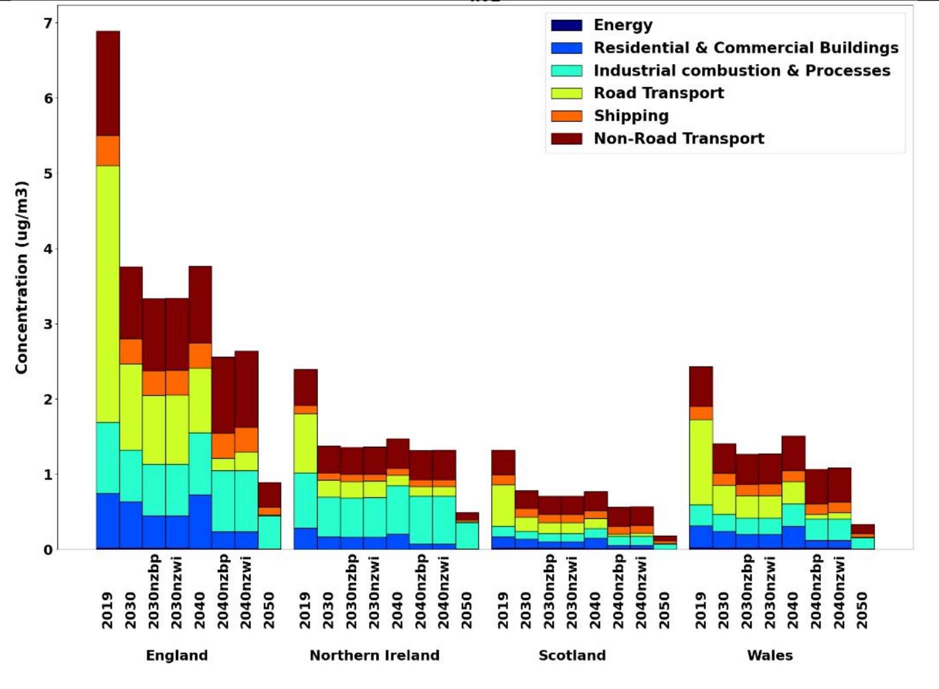

b) $PM_{2.5}$

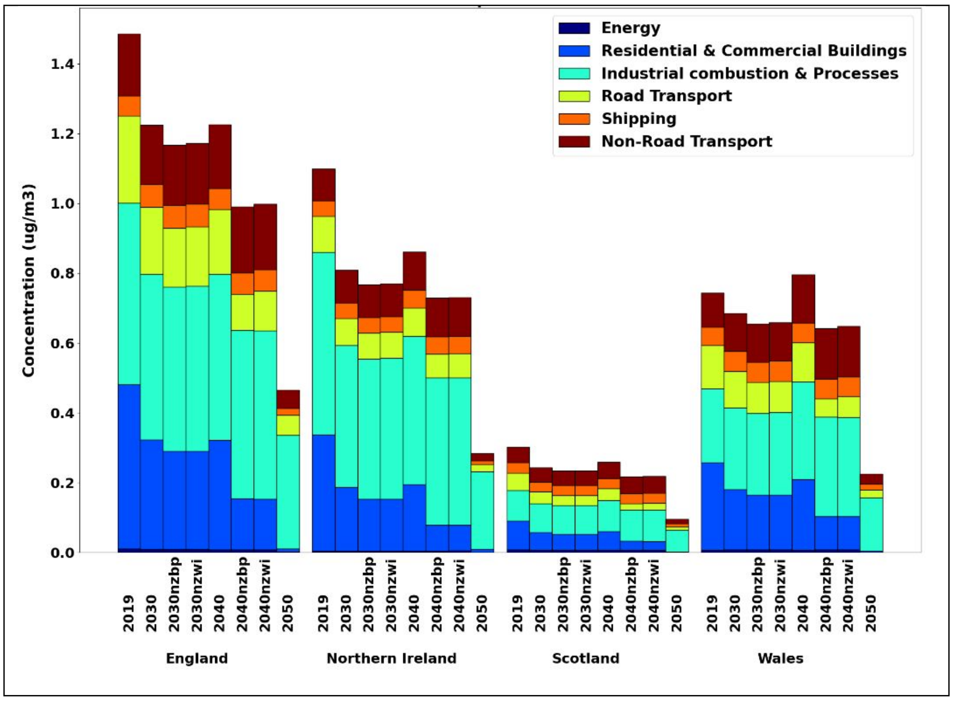

Figure S22 Source apportionment for each sector by country and scenario, NO_2 (a), and $PM_{2.5}$ (b).

Section S5 *Indoor air pollution modelling*

Indoor pollution can be released directly from indoor emissions (e.g. from cooking and heating), resuspension or ingress of outdoor air. The exchange of air pollution between indoor and outdoor environments is regulated by home ventilation, predominantly influenced by the characteristics of the home and outdoor/indoor climatic conditions. The impact of the NZ policies on indoor air pollution exposure was assessed through three main pathways: 1) Alterations in outdoor air quality and meteorology, 2) Modifications to home characteristics (specifically, insulation and mechanical ventilation), and 3) Adjustments in indoor sources, specifically the removal of fossil fuels such as the transition from gas to electricity for cooking. The indoor CONTAM model (41) was optimised and then evaluated with six-months of personal exposure measurements conducted within

a representative three bed terraced house in London as shown in Figure S23 (FAC2 and r were 0.49-0.83 and 0.65-0.76 for daily $PM_{2.5}$ and NO_2 concentrations, respectively). Subsequently, the indoor model was then employed to forecast indoor exposure to air pollution across varied scenarios of residential ventilation conditions including window settings (open, closed or open following cooking schedule), cook hood operation (on or off), mechanical ventilation system status (on or off) and presence or absence of wall insulation (yes or no), indoor sources (specifically, cooking by boiling using gas or electricity) and external outdoor conditions (air quality and meteorology for the years 2019 and 2040).

To assess the impacts of NZ policies on indoor air quality, we utilised the well-established multizone airflow and contaminant transport modelling software, CONTAM (version 3.4) (41). The CONTAM model, developed and maintained by the National Institute of Standards and Technology (NIST), is an extensively utilized simulation tool for indoor air quality research. To predict and forecast indoor exposure to air pollution, the CONTAM model relies on five main inputs: 1) Outdoor air quality and weather conditions; 2) Building characteristics (i.e., building archetypes and ventilation systems); 3) Indoor climate (temperature and humidity); 4) Indoor dynamics of contaminants (emission sources, loss rates); and 5) Occupant behaviour which determines ventilation settings, indoor source generation, and time spent in different rooms. More details of the CONTAM model inputs is presented in Tables S8.

According to the UK's energy performance certificate (EPC) database, (need a link) terraced houses constitute 36% of the overall London property types, with mid-terrace structures comprising 43% of the built form within this category, and representing 1.5 million homes. A three-bedroom mid-terraced dwelling, having indoor measurements was therefore chosen for the air pollution assessment using CONTAM.

In our study, we predicted indoor exposure to $PM_{2.5}$ and NO_2 at home for the year 2019 and forecast it for 2040, for the BAU and BNZP scenarios using outdoor air quality and meteorological predictions from CMAQ-urban

and WRF. 2019 model evaluation is shown in Figure S24. We then tested different scenarios, including changes to airtightness and ventilation resulting from home insulation and heat pump installation, and indoor sources altered by transitioning from gas to electricity for cooking. We assumed that cooking methods, natural ventilation, time spent at home, and the indoor dynamics of indoor pollutants remain unchanged between 2019 and 2040. We calculated human indoor exposure concentrations by considering the time individuals spent in different rooms of the house, estimated using data from the Real-time assessment of community transmission REACT surveys (42).

Table S8 Input parameter for CONTAM model

Input parameters		Descriptions	References
**Outdoor air quality and weather**	Air pollutants ($PM_{2.5}$ and NO_2)	Average concentrations extracted from CMAQ-Urban for all London's house address	CMAQ-Urban models

conditions in 2019 and 2040	Weather	Averaged windspeed/directions, temperature and humidity extracted from WRF model for London	Our WRF models
House characteristics	House types	Two-storey terraced houses (3 beds)	Measurements
	Floor areas/height	Kitchen/living room (30m^2); main bedroom (12.2m^2); other beds (6m^2); Floor height (2.7m)	Measurements
	Energy Performance Certificate	D in 2019 and expected to C or above in 2040	England & Wales EPC database
	Airtightness	11.3 m^3/(m^2.h)@50 Pa in 2019 and expected to 5 m^3/(m^2.h) after house insulation	Estimated values by authors & UK home standards (document F)
House ventilation settings	Cooking Extractor	30 l/s (Ventilate cooking emissions outside)	UK home standards (document F)
	MVHR systems	Continuous supply/return air: 13l/s for kitchen/main bedroom, 6l/s for additional bedroom, 8l/s for bathroom	UK home standards (document F)
Indoor climates	Temperature	19.2 ± 1.1 ^{o}C	Measurements
	Relative Humidity	53.5 ± 4.2%	
NO2 indoor dynamics	Loss rates	0.75/h (summer), 0.50/h (winter)	
PM2.5 indoor dynamics	Penetration factors	1 (opened windows/ doors), 0.86 (closed windows/doors)	(43)
	Deposition rates	0.32/h (summer), 0.24/h (winter)	
Cooking emission rates	NO2	1.0 mg/min (gas boiling), 0 mg/min (electricity boiling)	(44)
	PM2.5	0.35 mg/min (gas boiling), 0.22 mg/min (electricity boiling)	
Occupants' patterns	Time spent at homes	Weekday/Weekend: 4.8/5.6h (living room); 2.1/2.3h (kitchen and dinning room); 8.0/8.3h (bedroom)	Our REACT surveys
	Cooking times	7.00-7.20 am, 12.00-12.30 pm (only Weekend), and 18.00-18.40	Assumption
	Window and MVHR schedules	7.00-8.00 am, 12.00-1.00 pm (only Weekend), and 18.00-19.00	Assumption

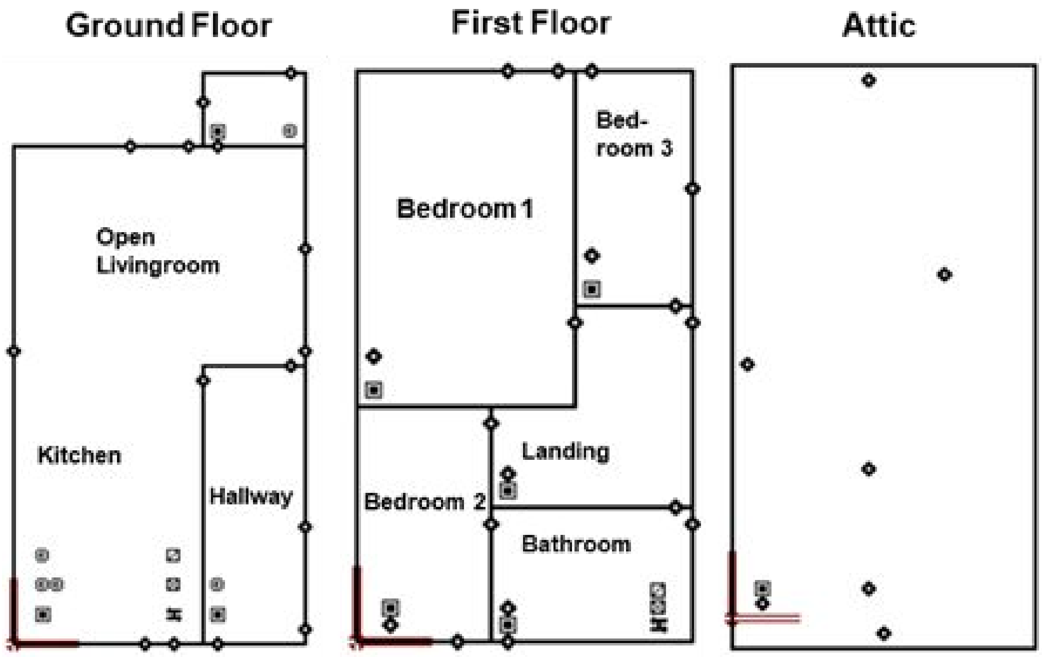

Figure S23. Floor plan for our test house

In our study, we predict indoor exposure at home for the year 2019 and forecast it for 2040, considering the BAU and BNZP scenarios for outdoor air quality. The differences in input parameters between these scenarios include variations in outdoor air quality and climate, airtightness and ventilation resulting from home insulation and heat pump installation, and indoor sources altered by transitioning from gas to electricity. We specifically examined the effects on indoor exposure of a switch between gas and electric cooking, assumed that there were no emissions related to what you cook, and that the use of natural ventilation, time spent at home, and the indoor dynamics of indoor pollutants remain unchanged between 2019 and 2040.

We calculated human indoor exposure concentrations by considering the time weighted average that individuals spend in different rooms of the house. The time spent in different rooms was estimated using an survey of indoor environmental data from 16,000 REACT households (42) and focused on exposure to $PM_{2.5}$ and NO_2. To demonstrate that the CONTAM model represents indoor air pollution well, model evaluation against sensor data taken from the COPE study is shown in Figure S24.

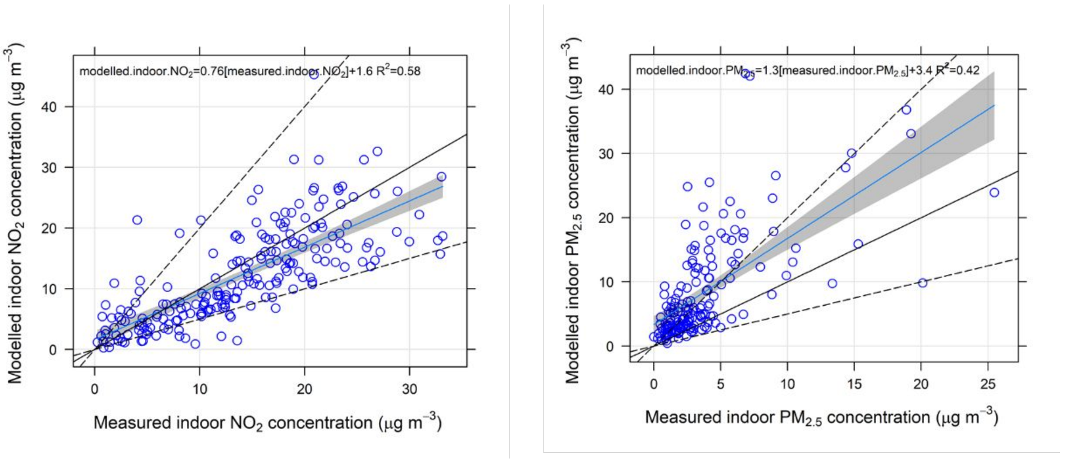

Figure S24 Model evaluation for daily NO_2 and $PM_{2.5}$

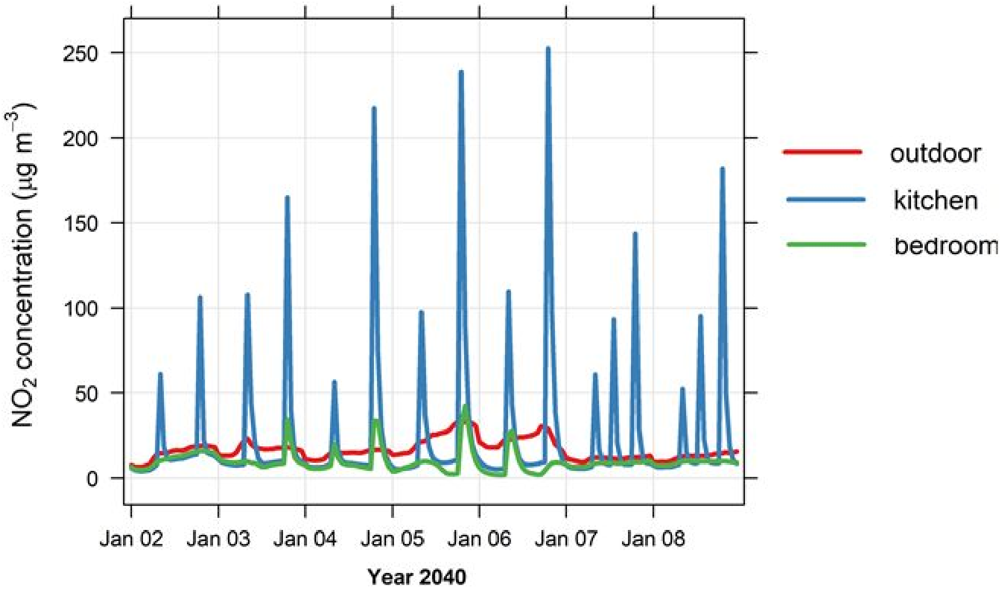

Figure S25 Estimated hourly exposure to NO_2 in the first 10 days of 2040 in a house using gas for cooking with limited ventilation.

Figure S26 shows the relative change of indoor exposure to $PM_{2.5}$ and NO_2 under different scenarios for homes in 2019 and 2040, compared with their outdoor concentrations measured in 2019. The average outdoor concentrations of $PM_{2.5}$ and NO_2 at homes in London during 2019 were 10.4 and 23.4 µg m^{-3}, respectively. Projections of outdoor concentrations for the year 2040 reduce to 7.8 µg m^{-3} (24.7%) for $PM_{2.5}$ and 14.9 µg m^{-3} (36.5%) for NO_2 under BAU, further decreasing to 7.2 and 12.1 µg m^{-3} (30.8% and 48.3%) under the BNZP scenario. Concerning indoor exposure to air pollution, in the absence of indoor sources, the home can shield individuals from outdoor air pollution, resulting in reductions of 38.3% and 44.9% for $PM_{2.5}$ and NO_2, in 2019.

In this case, the enhancement of home insulation by 2040 could further diminish indoor exposure to $PM_{2.5}$ and NO_2 by 11.1% and 9.7%, respectively.

However, when indoor sources such as cooking are present, greater air tightness through insulation limiting indoor to outdoor air exchange, contributes to increased indoor air pollution exposure. Where gas is utilised for cooking and there is no mechanical ventilation in an insulated home by 2040, indoor exposure to $PM_{2.5}$ and NO_2 was predicted to increase by 23.5% and 10.4%, in comparison to the levels observed in 2019. Notably, the hourly exposure to NO_2 could occasionally exceed the hourly standard of 200 μg m^{-3}, as shown in Figure S25. This emphasizes the need to consider indoor emission source control, enhancing natural ventilation, and implementing mechanical ventilation, particularly in houses that are relatively airtight (Ng et al., 2018). Home ventilation by maintaining permanently open windows is impractical and permits the ingress of NO_2 from outside, so the use of ventilation during cooking emerges as the most effective alternative. Furthermore, as depicted in Figure S25, the findings indicate that the transition from gas to electricity for cooking yields significant advantages in mitigating indoor exposure to air pollutants, particularly for NO_2 with a reduction of more than 80% by 2040. In identical ventilation conditions, indoor exposure to $PM_{2.5}$ using electricity for cooking (boiling method) (ref) is found to be lower by approximately 0.68 μg m^{-3} (9.2%), compared to utilising gas cooking. In this example, mechanical ventilation with a filtration system has the potential to further decrease indoor exposure to $PM_{2.5}$, albeit with a significant increase in the infiltration of outdoor NO_2.

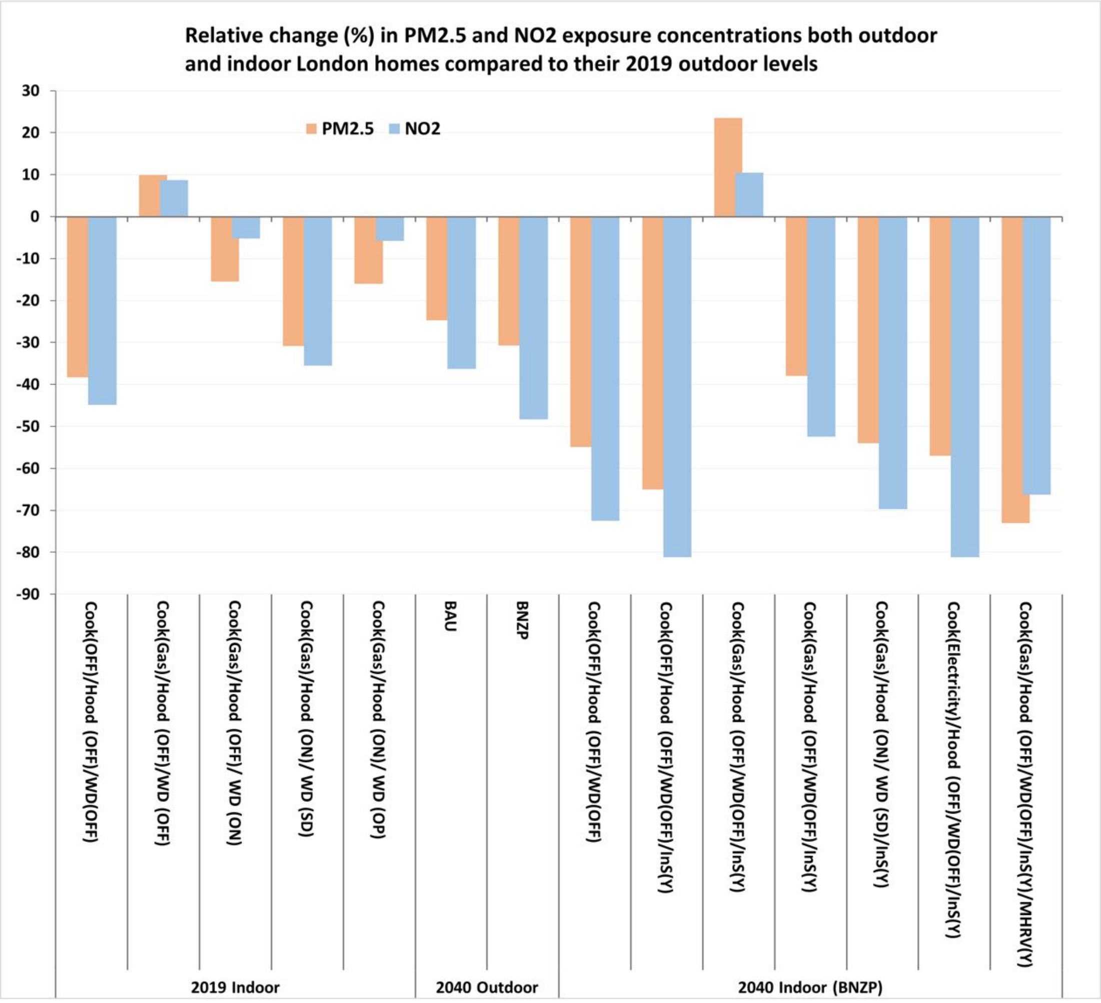

Figure S26 Relative changes (%) in $PM_{2.5}$ and NO_2 exposure in different outdoor conditions and home ventilation scenarios compared to their 2019 outdoor exposure.

Note that WD: Windows; Ins: Insulation; MVHR: Mechanical Ventilation Heat Recovery system; Y: Yes

References

1. Solazzo, E.; Bianconi. R.; Hogrefe. C.; Curci. G.; Tuccella, P.; Alyuz, U.; Balzarini, A.; Baró, R.; Bellasio, R.; Bieser, J.; Brandt, J.; Christensen, J. H.; Colette, A.; Francis, X.; Fraser, A.; Vivanco, M. G.; Jiménez-Guerrero, P.; Im, U.; Manders, A.; Nopmongcol, U.; Kitwiroon, N.; Pirovano, G.; Pozzoli, L.; Prank, M.; Sokhi, R. S.; Unal, A.; Yarwood, G.; Galmarini, S. (2017). Evaluation and error apportionment of an ensemble of atmospheric chemistry transport modeling systems: multivariable temporal and spatial breakdown. Atmos Chem Phys. 2017;17(4):3001-54. https://doi.org/10.5194/acp-17-3001-2017
2. EC (2021). European Commission. The second clean air outlook. Brussels, 8.1.2021 COM (2021) 3 final. https://climate.ec.europa.eu/eu-action/climate-strategies-targets/2050-long-term-strategy_en. (Accessed November 15, 2023)
3. NAEI (2019). UK National emission inventory. https://naei.beis.gov.uk/data/data-selector?view=greenhouse-gases. (Accessed May 20, 2024)
4. BEIS (2019). Energy and Emission Projections (EEP) 2018 (April 2019). https://assets.publishing.service.gov.uk/government/uploads/system/uploads/attachment_data/file/794590/updated-energy-and-emissions-projections-2018.pdf. (Accessed December 5, 2023).
5. LAEI (2019). London atmospheric emissions inventory 2019. https://data.london.gov.uk/dataset/london-atmospheric-emissions-inventory--laei--2019. (Accessed May 18, 2024)

6. LES (2018). London environment strategy 2018. https://www.london.gov.uk/sites/default/files/london_environment_strategy_0.pdf. (Accessed May 20, 2024)
7. CCC (2020a). Committee on Climate Change. The Sixth Carbon Budget report, 9 December 2020. https://www.theccc.org.uk/publication/sixth-carbon-budget/. (Accessed November 14, 2023)
8. Hicks, W., Green, D. C., & Beevers, S. (2023). Quantifying the change of brake wear particulate matter emissions through powertrain electrification in passenger vehicles. Environmental Pollution, 336, 122400.
9. Dajnak, D., Assareh, N., Kitwiroon, N., Beddows, A. V., Stewart, G. B., Hicks, W., & Beevers, S. D. (2023). Can the UK meet the World Health Organization PM2.5 interim target of 10 μg m-3 by 2030?. Environment International, 108222.
10. Emisia (2023). COPERT v5.4 Available from https://www.emisia.com/utilities/copert/ (Accessed November 28, 2023)
11. DEPC (2022a). Domestic Energy Performance Certificate for England and Wales https://epc.opendatacommunities.org/login. (Accessed 20 May, 2024)
12. DEPC (2022b). Domestic Energy Performance Certificate for Scotland. https://statistics.gov.scot/data/domestic-energy-performance-certificates. (Accessed 20 May, 2024)
13. Sub-regional fuel poverty status for England and Wales 2019 data (2021). https://www.gov.uk/government/statistics/sub-regional-fuel-poverty-data-2021 (Accessed 10 May, 2024)

14. Scottish Index of Multiple Deprivation (2020). https://www.gov.scot/collections/scottish-index-of-multiple-deprivation-2020/ (Accessed 10 May, 2024)

15. Northern Ireland Multiple Deprivation Measure (2017). https://www.nisra.gov.uk/statistics/deprivation/northern-ireland-multiple-deprivation-measure-2017-nimdm2017. (Accessed 10 May, 2024)

16. Estimates of heat use, (2014). Special feature – Estimates of heat use in the United Kingdom in 2013, December 2014. https://assets.publishing.service.gov.uk/government/uploads/system/uploads/attachment_data/file/386858/Estimates_of_heat_use.pdf. (Accessed 10 November, 2023)

17. BEIS (2021). Opportunity areas for district heating networks in the UK National Comprehensive Assessment of the potential for efficient heating and cooling, September 2021. https://www.gov.uk/government/publications/opportunity-areas-for-district-heating-networks-in-the-uk-second-national-comprehensive-assessment. (Accessed 2 March, 2024)

18. Element energy (2021). Development of trajectories for residential heat decarbonisation to inform the Sixth Carbon Budget A study for the Committee on Climate Change, April 2021. https://www.theccc.org.uk/publication/development-of-trajectories-for-residential-heat-decarbonisation-to-inform-the-sixth-carbon-budget-element-energy/ (Accessed 25 May, 2024)

19. FES, (2022). Future Energy Scenarios. https://www.nationalgrideso.com/future-energy/future-energy-scenarios. (Accessed 10 November, 2023)

20. MAPS Viewer Portal, 2022. https://mapsviewerportal.com/. (Accessed 20 December, 2023)

21. Hy4Heat Hydrogen-Ready Wall-Mounted Gas Boilers (2019). Worcester Bosch. https://static1.squarespace.com/static/5b8eae345cfd799896a803f4/t/616d78c680bd847c4efade9a/1634564294494/Bosch+HyLife+.pdf. (Accessed 15 December, 2023)

22. CCC (2020b). Committee on Climate Change. The sixth carbon budget methodology report. https://www.theccc.org.uk/wp-content/uploads/2020/12/The-Sixth-Carbon-Budget-Methodology-Report.pdf. (Accessed 26 November, 2023)
23. CCC (2020c). Climate Change Committee. The Sixth Carbon Budget report on Buildings. https://www.theccc.org.uk/wp-content/uploads/2020/12/Sector-summary-Buildings.pdf. (Accessed 26 November, 2023)
24. Mlawer, E. J., Taubman, S. J., Brown, P. D., Iacono, M. J., & Clough, S. A. (1997). Radiative transfer for inhomogeneous atmospheres: RRTM, a validated correlated-k model for the longwave. Journal of Geophysical Research: Atmospheres, 102(D14), 16663-16682.
25. Dudhia, J. (1989). Numerical study of convection observed during the winter monsoon experiment using a mesoscale two-dimensional model. Journal of Atmospheric Sciences, 46(20), 3077-3107.
26. Jiménez, P. A., Dudhia, J., González-Rouco, J. F., Navarro, J., Montávez, J. P., & García-Bustamante, E. (2012). A revised scheme for the WRF surface layer formulation. Monthly weather review, 140(3), 898-918.
27. Chen, F., & Dudhia, J. (2001). Coupling an advanced land surface–hydrology model with the Penn State–NCAR MM5 modeling system. Part I: Model implementation and sensitivity. Monthly weather review, 129(4), 569-585.
28. Hong, S. Y., Noh, Y., & Dudhia, J. (2006). A new vertical diffusion package with an explicit treatment of entrainment processes. Monthly weather review, 134(9), 2318-2341.
29. Kain, J. S., & M. Fritsch, (1993) Convective parameterization for mesoscale models: The Kain–Fritsch scheme. The Representation of Cumulus Convection in Numerical Models, Meteor. Monogr., No. 46, Amer. Meteor. Soc., 165–170.

30. Yarwood, G., Jung, J., Whitten, G. Z., Heo, G., Mellberg, J., & Estes, M. (2010). Updates to the Carbon Bond mechanism for version 6 (CB6). In 9th Annual CMAS Conference, Chapel Hill, NC (pp. 11-13)

31. Pleim, J., Venkatram, A., & Yamartino, R. (1984). ADOM/TADAP Model Development Program, the Dry Deposition Module; ERT document P-B980-520; Prepared for Ontario Ministry of the Environment and Applied Engineering and Science, Canada by ERT. Inc., Concord, MA.

32. Pleim, J., & Ran, L. (2011). Surface flux modeling for air quality applications. Atmosphere, 2(3), 271-302.

33. Fahey KM, Sareen N, Carlton AG, Hutzell WT, and Luecken DJ: Regional impacts of extending inorganic and organic cloud chemistry with AQCHEM-KMT, in: 16th Annual CMAS Conference, Chapel Hill, North Carolina, USA, poster; no. 17, 2017b

34. Appel, K. W., Bash, J. O., Fahey, K. M., Foley, K. M., Gilliam, R. C., Hogrefe, C., Hutzell, W. T., Kang, D., Mathur, R., Murphy, B. N., Napelenok, S. L., Nolte, C. G., Pleim J. E., Pouliot, G. A., Pye, H .O .T., Ran, L., Roselle, S. J., Sarwar, G., Schwede, D. B., Sidi, F. L., Spero, T. L., & Wong, D. C. (2021). The Community Multiscale Air Quality (CMAQ) model versions 5.3 and 5.3. 1: system updates and evaluation. Geoscientific model development, 14(5), 2867-2897.

35. Sandu, A., Verwer, J. G., Blom, J. G., Spee, E. J., Carmichael, G. R., & Potra, F. A. (1997). Benchmarking stiff ODE solvers for atmospheric chemistry problems II: Rosenbrock solvers. Atmospheric environment, 31(20), 3459-3472.

36. Colella, P., & Woodward, P. R. (1984). The piecewise parabolic method (PPM) for gas-dynamical simulations. Journal of computational physics, 54(1), 174-201.

37. Met Office (2006). MIDAS: UK Hourly Weather Observation Data. NCAS British Atmospheric Data Centre. https://catalogue.ceda.ac.uk/uuid/916ac4bbc46f7685ae9a5e10451bae7c. (Accessed November 21, 2023)

38. Defra (2022). Department for Environment, Food and Rural Affairs. Automatic Urban and Rural Network (AURN). https://uk-air.defra.gov.uk/networks/network-info?view=aurn (Accessed December 2, 2023)

39. LAQN (2022). London Air Quality Network. https://www.londonair.org.uk/LondonAir/Default.aspx. (Accessed December 10, 2023)

40. Ricardo (2022) Ricardo Energy & Environment. Air Quality England. http://www.airqualityengland.co.uk/. (Accessed December 10, 2023)

41. Dols, W. S., & Polidoro, B. (2015). CONTAM user guide and program documentation: version 3.2. US Department of Commerce, National Institute of Standards and Technology.

42. Riley, S., Atchison, C., Ashby, D., Donnelly, C. A., Barclay, W., Cooke, G. S., Ward, H., Darzi, A., Elliot, P., & REACT Study Group. (2020). Real-time assessment of community transmission (REACT) of SARS-CoV-2 virus: study protocol. Wellcome Open Research, 5.

43. Vu, T. V., Stewart, G. B., Kitwiroon, N., Lim, S., Barratt, B., Kelly, F. J., Thompson, R., Smith, R. B., Toledano, M. B., Beevers, S. D. (2022). Assessing the contributions of outdoor and indoor sources to air quality in London homes of the SCAMP cohort. Building and Environment, 222, 109359.

44. Lebel, E. D., Finnegan, C. J., Ouyang, Z., & Jackson, R. B. (2022). Methane and NO x emissions from natural gas stoves, cooktops, and ovens in residential homes. Environmental science & technology, 56(4), 2529-2539.

45. Sun, L., & Wallace, L. A. (2021). Residential cooking and use of kitchen ventilation: The impact on exposure. Journal of the Air & Waste Management Association, 71(7), 830-843
